# Cardiovascular disease mortality in a Central-West Brazilian capital: A spatial, temporal, and ecological analysis

**DOI:** 10.1371/journal.pone.0359337

**Published:** 2026-09-29

**Authors:** João Vitor Barrio, Rafael Santana Batista Cabral, Sonia Regina Maciel Rodrigues da Silva, Audêncio Victor, Maria Elizabeth Araújo Ajalla, Cláudia Du Bocage Santos-Pinto, Everton Falcão de Oliveira

**Affiliations:** 1 Faculdade de Medicina, Universidade Federal de Mato Grosso do Sul, Campo Grande, Mato Grosso do Sul, Brazil; 2 Programa de Pós-Graduação em Doenças Infecciosas e Parasitárias, Universidade Federal de Mato Grosso do Sul, Campo Grande, Mato Grosso do Sul, Brazil; 3 Faculty of Epidemiology and Population Health, London School of Hygiene and Tropical Medicine, London, United Kingdom; Oklahoma State University, UNITED STATES OF AMERICA

## Abstract

In Brazil, regional disparities in cardiovascular disease (CVD) mortality persist, with no population-based study having examined CVD mortality at the municipal level in the Central-West region. This study analysed the epidemiological profile, temporal trends, spatial distribution, and socioeconomic correlates of CVD mortality in Campo Grande, Mato Grosso do Sul, Brazil (2013–2022). A population-based ecological study used data from the Brazilian Mortality Information System. Five CVD groups were analysed: ischaemic heart disease (IHD), heart failure (HF), arrhythmias (ARR), cardiomyopathies (CMP), and inflammatory heart diseases (INF). Temporal trends were assessed using Poisson, Prais-Winsten, and Joinpoint regression. Spatial distribution was examined through Kernel Density Estimation and Local Indicators of Spatial Association (LISA). Socioeconomic correlates were assessed using Spearman’s correlation with neighbourhood-level indicators. Of 59,392 registered deaths, 8,148 (13.7%) were CVD-related. IHD was the dominant cause (79.5%), with males accounting for 59.3% of deaths (IRR 1.59; 95% CI 1.46–1.74) and individuals aged ≥60 years representing 75.2%. Overall crude CVD mortality increased by 26.1% over the study period, with an estimated mean annual increase of 2.5% (APC + 2.5%/year; 95% CI 1.7–3.3; p < 0.001) and significant acceleration after 2019. A striking proportion of IHD deaths occurred outside hospital settings (67.3%), with home deaths alone surpassing hospital deaths (43.9% vs. 32.7%). Spatial analysis identified significant high-high mortality clusters in central-western neighbourhoods and low-low clusters in eastern neighbourhoods (13 and 5 neighbourhoods, respectively; Global Moran’s I = 0.555, p < 0.001), with cluster patterns varying by disease group. Socioeconomic correlates showed a consistent pattern across most CVD groups, with higher mortality in more socioeconomically advantaged neighbourhoods, except for inflammatory heart disease, which showed the opposite pattern. This first municipal-level CVD mortality analysis in Central-West Brazil identified significant spatiotemporal heterogeneity and a high burden of out-of-hospital IHD deaths, providing actionable evidence for geographically targeted cardiovascular prevention strategies.

## Introduction

Cardiovascular diseases (CVD) are the leading cause of death globally, accounting for approximately 19.1 million deaths in 2020 and 32% of all deaths worldwide [1]. CVDs are strongly associated with social determinants of health, disproportionately affecting socioeconomically disadvantaged populations, and in low- and middle-income countries undergoing epidemiological transition, limited access to health services and poor continuity of care sustain persistently high rates of CVD mortality [2].

In Brazil, CVD has consistently ranked as the primary cause of mortality, with approximately 400,000 deaths per year attributed to diseases of the circulatory system [3]. National studies have documented declining trends in age-standardised CVD mortality, attributed to improvements in risk factor control, expanded primary health care, and advances in emergency care [4,5]. However, this overall decline masks important regional heterogeneities: the Central-West and North regions have shown persistently higher or stagnating rates compared with the South and Southeast [6,7]. In 2021, the age-standardised CVD mortality rate in Mato Grosso do Sul (170.9 per 100,000) exceeded the national average (162.2 per 100,000) [3]. At the municipal level, large municipalities (>300,000 inhabitants) in the Central-West region recorded lower CVD mortality than the national average for equivalent municipalities in 2016–2018 (158 vs. 178 per 100,000) [7], although this reduction has been shown to be slower and more heterogeneous than in the South and Southeast [7]. Despite this regional heterogeneity, population-based studies characterising the spatial and temporal dynamics of CVD mortality at the municipal level remain scarce in the Central-West region, limiting the evidence base needed to inform regionally-tailored public health interventions in a part of the country marked by pronounced socioeconomic and epidemiological heterogeneity.

Campo Grande, the state capital of Mato Grosso do Sul, offers a particularly relevant setting for this investigation, given its location in a border state, intense demographic flows, and rapid urbanisation from the 2000s onward [8], factors previously associated with increased cardiovascular risk in Brazil through lifestyle changes, rising exposure to risk factors, and disparities in healthcare access [4]. This border position is additionally associated with significant population mobility, which may further compound heterogeneity in cardiovascular risk exposure and healthcare-seeking patterns. This period of urbanisation coincided with the expansion of primary health care services [9], which, within the Unified Health System (*Sistema Único de Saúde*, SUS), included public policies to register, monitor, and treat patients with hypertension and diabetes mellitus, such as the HIPERDIA program.

Although national studies have documented spatiotemporal patterns of CVD mortality across Brazilian states and municipalities [7,10], these analyses have not examined the social determinants underlying such patterns at the local level, a critical gap given the marked socioeconomic heterogeneity observed across the country’s regions. To date, no population-based study has investigated the spatial and temporal dynamics of CVD mortality in any municipality of the Central-West region. Therefore, this study aimed to analyze the epidemiological profile, spatial distribution, socioeconomic determinants, and temporal patterns of CVD mortality in Campo Grande, Mato Grosso do Sul, Brazil, over a ten-year period.

## Methods

### Ethics statement

This study was approved by the Research Ethics Committee of the Federal University of Mato Grosso do Sul (CAAE: 74807523.7.0000.0021). Informed consent was waived because this study used administrative de-identified data, analysed under strict data security procedures, in accordance with the Brazilian General Data Protection Law [11]. All methods were performed in accordance with the Declaration of Helsinki.

### Study design, setting, and period

We conducted a population-based ecological study in Campo Grande, Central-West Brazil, using mortality data from 2013 to 2022. The study was designed in 2023 to analyse a 10-year time series; as 2022 was the most recent year with available data at the time of analysis, the study period was defined accordingly. The data were received from the Campo Grande Municipal Health Department in November 2024 and were analysed between January and November 2025. No sensitive information enabling individual identification was accessed at any stage of data collection or analysis.

Campo Grande has a territorial area of 8,118.4 km² and is administratively divided into 74 neighbourhoods, which served as the units of analysis for the ecological component of the study. The municipality had an estimated population of 898,100 inhabitants in 2022, with a predominance of women (52.6%) and evidence of population ageing, characterized by a narrowing of the base and widening of the top of the age pyramid, according to the Brazilian Institute of Geography and Statistics [8].

### Data sources

Mortality data were obtained from the Mortality Information System (*Sistema de Informação sobre Mortalidade*, SIM) of the Brazilian Ministry of Health, a nationwide vital registration system that compiles information from all death certificates in the country. The SIM has achieved coverage exceeding 95% and is considered to have acceptable data quality for epidemiological analyses in Brazilian state capitals [12]. Specifically, Campo Grande has consistently ranked among the state capitals with the lowest proportion of ill-defined causes of death in the national Garbage Codes Monitoring Panel (DAENT/SVSA, Brazilian Ministry of Health) [13] and presents official sub-registration and subnotification rates for deaths of 0.20% and 0.09%, respectively, both well below national averages (3.40% and approximately 1%) [14] Each record includes demographic, clinical, and geographic information derived from a standardised death certificate.

Population denominators were obtained from the Brazilian Institute of Geography and Statistics (*Instituto Brasileiro de Geografia e Estatística*, IBGE), using intercensal estimates based on geometric growth projections for 2013–2021 and data from the 2022 Population Census for the final study year. Sex-stratified population estimates were used to calculate sex-specific mortality rates.

Neighbourhood-level sociodemographic data were obtained from the Municipal Department of Environment and Urban Planning of Campo Grande (*Agência Municipal de Meio Ambiente e Planejamento Urbano*, PLANURB) [15]. These composite indices (Education, Poverty/Income, Environmental Sanitation, Housing Quality, and Urban Quality of Life) were based on the 2010 Population Census, as neighbourhood-level data for these indicators from the 2022 Census were not yet available at the time of analysis.

For neighbourhood-level population denominators, annual population estimates for each of the 74 neighbourhoods (2013–2022) were obtained by geometric interpolation between the 2010 and 2022 Population Censuses, applying neighbourhood-specific growth rates [16]. Population totals aggregated across the 74 urban neighbourhoods represented 94.4–98.4% of the municipality’s official total population in each year, with the residual attributable to rural and peri-urban areas outside the formal neighbourhood boundaries; neighbourhood-level population estimates were therefore proportionally calibrated (pro-rated) to match official annual municipal population totals, obtained from IBGE’s intercensal population estimates [17].

Neighbourhood boundary data for Campo Grande were obtained from the Municipal Geospatial System (*Sistema Municipal de Geoprocessamento*, SIMGEO) in shapefile format, made available through the official geospatial data repository of the Campo Grande Municipal Government [18].

### Participants and case definition

The study population comprised all residents of Campo Grande whose deaths were registered in the SIM between 1 January 2013 and 31 December 2022. The study population comprised all residents of Campo Grande whose deaths were registered in the SIM between 1 January 2013 and 31 December 2022. Death records were initially extracted by the Campo Grande Municipal Health Department based on the place of occurrence of death; residency status was subsequently verified using the municipality of residence field recorded on the death certificate, and only deaths of individuals residing in Campo Grande were retained for analysis, resulting in the exclusion of 1,644 records corresponding to non-residents. Deaths were included if the underlying cause of death was classified within Chapter IX of the International Classification of Diseases, 10th Revision (ICD-10), corresponding to diseases of the circulatory system, with a focus on cardiac conditions.

Deaths were further grouped into five mutually exclusive categories based on three-character ICD-10 codes: (i) ischaemic heart disease (I20–I25); (ii) heart failure and other cardiac conditions (I50–I52); (iii) cardiac arrhythmias and conduction disorders (I44–I49); (iv) cardiomyopathies (I42–I43); and (v) inflammatory heart diseases (I30–I41). Valvular heart diseases (I05–I09, I34–I37) were initially considered as a separate category but were excluded due to the absence of deaths classified under these codes as the underlying cause during the study period.

No records were excluded due to missing or incomplete information; missing data for each variable were instead quantified and are reported in the characterisation of the study population (Table 1). Duplicate records were considered highly unlikely given that death certificates in Brazil are serialised documents whose issuance is centrally controlled by the Ministry of Health; no duplicate records were identified in the dataset.

**Table 1:** Clinical and epidemiological characteristics of cardiovascular deaths by disease group, Campo Grande, MS, Brazil, 2013–2022.

Characteristic	Overall N=8,148	Ischaemic heart disease (IHD) N=6,476 (79.5%)	Heart failure (HF) N=558 (6.8%)	Arrhythmias (ARR) N=513 (6.3%)	Cardiomyopathies (CMP) N=419 (5.1%)	Inflammatory (INF) N=182 (2.2%)	p-value^2^
Sex							<0.001
Female	3,315 (40.7%)	2,516 (38.9%)	284 (50.9%)	267 (52.0%)	178 (42.5%)	70 (38.5%)	
Male	4,832 (59.3%)	3,959 (61.1%)	274 (49.1%)	246 (48.0%)	241 (57.5%)	112 (61.5%)	
Ignored	1 (0%)	1 (0%)	0 (0%)	0 (0%)	0 (0%)	0 (0%)	
Missing	0 (0%)	0 (0%)	0 (0%)	0 (0%)	0 (0%)	0 (0%)	
Age, years	70 (60–80)	69 (59–79)	76 (63-84)	79 (67–86)	71 (57–82)	63 (48–72)	<0.001
Age group, years							<0.001
0-19	21 (0.3%)	2 (0.0%)	5 (0.9%)	0 (0.0%)	10 (2.4%)	4 (2.2%)	
20-39	252 (3.1%)	159 (2.5%)	27 (4.8%)	19 (3.7%)	24 (5.7%)	23 (12.6%)	
40-59	1,736 (21.3%)	1,472 (22.7%)	76 (13.6%)	49 (9.6%)	91 (21.7%)	48 (26.4%)	
≥60	6,100 (74.9%)	4,816 (74.4%)	445 (79.7%)	440 (85.8%)	292 (69.7%)	107 (58.8%)	
Missing	39 (0.5%)	27 (0.4%)	5 (0.9%)	5 (1.0%)	2 (0.5%)	0 (0%)	
Race/Ethnicity							0.013
Asian	87 (1.1%)	61 (0.9%)	3 (0.5%)	11 (2.1%)	10 (2.4%)	2 (1.1%)	
Black	324 (4.0%)	244 (3.8%)	29 (5.2%)	23 (4.5%)	20 (4.8%)	8 (4.1%)	
Indigenous	18 (0.2%)	13 (0.2%)	1 (0.2%)	4 (0.8%)	0 (0.0%)	0 (0.0%)	
Mixed/*Pardo*	3,260 (40.0%)	2,667 (40.6%)	223 (40.0%)	175 (34.1%)	162 (38.7%)	73 (40.1%)	
White	4,452 (54.6%)	3,525 (54.4%)	301 (53.9%)	300 (58.5%)	227 (54.2%)	99 (54.4%)	
Missing	7 (0.1%)	6 (0.1%)	1 (0.2%)	0 (0.0%)	0 (0.0%)	0 (0.0%)	
Education level							<0.001
Illiterate	1,074 (13.2%)	837 (12.9%)	93 (16.7%)	89 (17.3%)	45 (10.7%)	10 (5.5%)	
Primary	3,893 (47.8%)	3,091 (47.7%)	270 (48.4%)	249 (48.5%)	205 (48.9%)	78 (42.9%)	
Secondary	2,082 (25.6%)	1,667 (25.7%)	119 (21.3%)	122 (23.8%)	110 (26.3%)	64 (35.2%)	
Higher education	626 (7.7%)	491 (7.6%)	45 (8.1%)	37 (7.2%)	32 (7.6%)	21 (11.5%)	
Missing	473 (5.8%)	390 (6.0%)	31 (5.6%)	16 (3.1%)	27 (6.4%)	9 (4.9%)	
Marital status							<0.001
Common-law	71 (0.9%)	53 (0.8%)	4 (0.7%)	4 (0.8%)	7 (1.7%)	3 (1.6%)	
Divorced/Separated	1,219 (15.0%)	1,016 (15.7%)	65 (11.6%)	60 (11.7%)	56 (13.4%)	22 (12.1%)	
Married	2.843 (34.9%)	2,286 (35.3%)	184 (33.0%)	165 (32.2%)	130 (31.0%)	78 (42.9%)	
Single	2,152 (26.4%)	1,733 (26.8%)	123 (22.0%)	125 (24.4%)	116 (27.7%)	55 (30.2%)	
Widowed	1,800 (22.1%)	1,348 (20.8%)	175 (31.4%)	157 (30.6%)	100 (23.9%)	20 (11.0%)	
Missing	63 (0.8%)	40 (0.6%)	7 (1.3%)	2 (0.4%)	10 (2.4%)	4 (2.2%)	
Place of death							<0.001
Home	3,116 (38.9%)	2,843 (43.9%)	97 (17.4%)	94 (18.3%)	100 (23.0%)	12 (6.6%)	
Hospital	3,317 (40.7%)	2,119 (32.7%)	416 (74.6%)	371 (72.3%)	249 (59.4%)	162 (89.9%)	
Other	359 (4.4%)	322 (5.0%)	12 (2.2%)	9 (1.8%)	15 (3.6%)	1 (0.5%)	
Other facility	1,167 (14.3%)	1,063 (16.4%)	32 (5.7%)	35 (6.8%)	31 (7.4%)	6 (3.3%)	
Public road	139 (1.7%)	129 (2.0%)	1 (0.2%)	4 (0.8%)	4 (1.0%)	1 (0.5%)	
Missing	0 (0%)	0 (0%)	0 (0%)	0 (0%)	0 (0%)	0 (0%)	

^1^ n (%); Median (Q1–Q3)

^2^ Pearson’s Chi-squared test; Kruskal-Wallis rank sum test; p-values from chi-squared test (categorical) or Kruskal-Wallis test (continuous).

IHD: ischaemic heart disease; HF: heart failure; ARR: arrhythmias; CMP: cardiomyopathies; INF: inflammatory heart diseases. Values are n (%) for categorical variables and median (IQR) for continuous variables.

### Variables

Individual-level data extracted from the SIM included: year of death; sex (male, female); age at death (in completed years); self-reported race/ethnicity as recorded on the death certificate (White, Mixed/Pardo, Black, Asian, Indigenous); marital status (married, single, widowed, divorced/separated, common-law); educational attainment (illiterate, primary, secondary, and higher education); place of death (hospital, home, other healthcare facility, public road, other); and the underlying cause of death coded according to ICD-10. Age was categorised into four epidemiological groups for descriptive analyses: 0–19 years (children and adolescents), 20–39 years (young adults), 40–59 years (adults), and ≥60 years (older adults). For age standardisation, nine finer age strata were used: 0–4, 5–14, 15–24, 25–34, 35–44, 45–54, 55–64, 65–74, and ≥75 years.

Neighbourhood-level aggregated variables were obtained from the PLANURB and included pre-calculated composite indices and neighbourhood-level individual indicators. The composite indices comprised: (i) the Education Index, reflecting educational attainment based on the proportion of literate individuals aged ≥10 years; (ii) the Poverty/Income Index, capturing economic conditions based on average household income and the proportion of households earning above one minimum wage; (iii) the Environmental Sanitation Index, measuring access to urban infrastructure, including piped water, sewage systems, and solid waste collection; (iv) the Housing Quality Index, assessing housing adequacy based on ownership status, structural conditions, and number of exclusive-use bathrooms; and (v) the Urban Quality of Life Index, a composite measure derived from the preceding indices summarising overall neighbourhood living conditions. Additional neighbourhood-level indicators included: access to sewage system, defined as the proportion of households connected to a sewage network; access to piped water supply, defined as the proportion of households with access to piped water; waste collection service, defined as the proportion of households with direct waste collection; average household occupancy, measured as the mean number of residents per household; and average monthly income, defined as the mean nominal monthly income of residents.

### Statistical analysis

#### Descriptive analysis.

The epidemiological profile of the study population was described using standard descriptive statistics. Categorical variables were summarised as frequencies and proportions (%), while age at death was described using the median and interquartile range (IQR) due to its non-normal distribution. Differences across disease groups were assessed using Pearson’s chi-squared test for categorical variables and the Kruskal-Wallis test for age. As pre-specified in the analysis plan, data completeness was evaluated by calculating the proportion of missing values for each variable.

Crude mortality rates were calculated for each year, disease group, and sex by dividing the number of deaths by the corresponding mid-year population estimate, expressed per 100,000 inhabitants. Age-standardised mortality rates (ASMR) were computed using the direct standardisation method, which applies age-specific mortality rates observed in Campo Grande to a standard reference population to remove the confounding effect of age structure differences across time [19]. The WHO World Standard Population (Segi–Doll modified weights) was used as the reference, with the following weights per 100,000: 0–4 years (8,860), 5–14 (17,020), 15–24 (17,020), 25–34 (13,580), 35–44 (13,580), 45–54 (12,800), 55–64 (10,440), 65–74 (7,160), and ≥75 (3,540). Age-specific population denominators were estimated by applying the age-group proportions from the 2010 Census of Campo Grande to the annual total population estimates. Similarly, as annual sex-specific population estimates are not published by IBGE at the municipal level, sex-specific denominators were estimated by applying the sex proportion from the 2010 Census of Campo Grande to the annual total population estimates.

#### Temporal trend analysis.

Three complementary statistical approaches were employed to assess temporal trends in cardiovascular mortality, each addressing different analytical assumptions:

(i) Poisson regression: Mortality counts for each year were modelled using generalised linear models (GLM) with a Poisson distribution and logarithmic link function [20]. The natural logarithm of the mid-year population was included as an offset term, effectively modelling the mortality rate. The centred year (year minus 2013) was included as the sole predictor, and the annual percentage change (APC) was calculated as APC = [exp(β) − 1] × 100, where β is the regression coefficient for the year variable. Ninety-five percent confidence intervals for the APC were derived from the profile likelihood confidence intervals of β. Overdispersion was assessed by computing the ratio of the residual deviance to the residual degrees of freedom; when this ratio exceeded 1.5, indicating that the variance exceeded the mean (a violation of the Poisson equidispersion assumption), negative binomial regression was fitted as a sensitivity analysis [20].
(ii) Prais-Winsten generalised least squares regression: Because time-series data of mortality rates may exhibit serial autocorrelation (i.e., the mortality rate each year is correlated with the rate in the preceding year), the Prais-Winsten method was employed to obtain autocorrelation-corrected regression estimates [21]. This method first estimates the first-order autocorrelation coefficient (ρ) from the residuals of an ordinary least squares (OLS) regression of the mortality rate on the centred year, then transforms both the dependent and independent variables using the Cochrane-Orcutt procedure (y* = y_t − ρy_{t−1})  and re-estimates the model. The resulting slope coefficient represents the average annual change in the mortality rate (per 100,000) after removing serial autocorrelation. Trends were classified as increasing (positive slope, p < 0.05), decreasing (negative slope, p < 0.05), or stationary (p ≥ 0.05).
(iii) Joinpoint regression: To identify inflection points (breakpoints) where the temporal trend changed significantly in slope, segmented linear regression was applied to the overall crude mortality rate series using the method of Muggeo [22]. This approach iteratively estimates the location of one or more breakpoints in a piecewise linear regression, partitioning the time series into segments with distinct slopes. The number of joinpoints was determined using the Bayesian Information Criterion. For each segment, the slope and its statistical significance were reported. This method has been widely used in cancer and cardiovascular epidemiology to detect changes in mortality trends [23].

#### Sex-specific analysis.

Sex-specific crude mortality rates were calculated using sex-stratified population denominators. To quantify the magnitude of sex differences, the incidence rate ratio (IRR) comparing male to female mortality was estimated from a Poisson regression model including centred year, sex (female as reference), and a year-by-sex interaction term, with the log of the sex-specific population as the offset. A significant interaction term (p < 0.05) would indicate that temporal trends differed between males and females. The male-to-female mortality ratio was also calculated for each disease group.

#### Spatial analysis.

All death records were geocoded in R (version 4.5.1) using the *tidygeocoder* package, with the ArcGIS World Geocoding Service as the sole geocoding provider, using a hierarchical address-matching strategy prioritising, in order: exact street address, street-level interpolation, neighbourhood centroid, and municipality centroid; 91.7% of records were matched to an exact street address (full geocoding precision breakdown presented in Supporting Information S1 Table). Geocoded points were then aggregated to neighbourhood-level counts in R using point-in-polygon procedures (*sf* package). For the small proportion of cases whose geocoded coordinates did not fall within any of the 74 official neighbourhood boundaries, neighbourhood assignment was corrected, where possible, using the self-reported neighbourhood field recorded independently on the death certificate at the time of registration, cross-validated against the official neighbourhood boundaries; cases that could not be resolved by either method, predominantly corresponding to genuinely rural or peri-urban addresses, were excluded from the neighbourhood-level spatial analysis (n = 69, 0.85% of confirmed residents), although retained in all non-spatial analyses. All spatial data handling, analysis, and mapping steps described below were conducted entirely in R (version 4.5.1).

Neighbourhood-level cardiovascular mortality rates were calculated as the number of deaths accumulated over the study period (2013–2022) divided by the corresponding neighbourhood person-years (the sum of the annual calibrated population estimates described in the “Data sources” section), expressed per 100,000 inhabitants. Because crude neighbourhood-level rates are subject to instability in areas with small case counts or low population, rates were additionally smoothed using a Besag-York-Mollié (BYM) Bayesian hierarchical model, implemented via the *CARBayes* package in R, with a Poisson likelihood, an offset for neighbourhood person-years, and spatially structured and unstructured random effects specified using the same Queen contiguity matrix described below. Convergence of the BYM model was assessed by comparing posterior estimates across independent MCMC runs with different random seeds; the correlation between smoothed rate estimates from two runs was consistently > 0.999, indicating stable convergence. Neighbourhood-level rates were not age-standardised, owing to the unavailability of age-stratified population data at the neighbourhood level.

Spearman’s rank correlation coefficient was used to assess the association between neighbourhood-level socioeconomic indicators and cause-specific CVD mortality rates, given the non-normal distribution of the data. Scatterplot matrices with correlation coefficients were generated to visualise overall patterns and pairwise relationships between variables, using the *PerformanceAnalytics* package in R.

Kernel Density Estimation (KDE) was applied to examine the spatial concentration of deaths across the five CVD subgroups. KDE is a non-parametric method that estimates the continuous spatial distribution of events, allowing the identification of high-density clusters without imposing predefined boundaries. KDE analyses were performed in R using the *spatstat* package, applying a Gaussian kernel with a fixed bandwidth of 800 metres and a spatial resolution of 50 metres per pixel. Two complementary approaches were used: a cumulative analysis covering the entire 10-year study period (2013–2022), presented in the main text, and a temporal analysis using five consecutive two-year periods (2013–2014, 2015–2016, 2017–2018, 2019–2020, and 2021–2022), which allowed assessment of changes in spatial concentration over time.

Spatial autocorrelation was assessed using both the Global Moran’s I and Local Indicators of Spatial Association (LISA). The Global Moran’s I quantifies the overall degree of spatial clustering across the study area, indicating whether similar mortality values tend to occur near each other. LISA identifies specific neighbourhoods that contribute to this clustering, classifying them as high–high, low–low, high–low, or low–high clusters, thereby revealing localised patterns that may be masked in global analyses. LISA and Global Moran’s I analyses were applied to BYM-smoothed mortality rates rather than crude rates, to reduce instability arising from small numbers of events in low-population neighbourhoods. LISA quadrants were classified based on deviations from the mean of the variable and its spatial lag. Both analyses were conducted entirely in R (packages: *sf*, *spdep*, and *tmap*), using a Queen contiguity matrix with row-standardised weights. Statistical significance for the Global Moran’s I was assessed using 999 Monte Carlo permutations. As with the KDE analyses, spatial autocorrelation was examined for the full study period and for each of the five two-year intervals (2013–2014, 2015–2016, 2017–2018, 2019–2020, and 2021–2022) to characterise temporal changes in spatial dependence across neighbourhoods.

As a complementary, exploratory analysis, we investigated the relationship between neighbourhood-level CVD mortality and socioeconomic covariates using a generalised additive model (GAM) with a space-time interaction term, allowing for non-linear associations that the Spearman correlation approach described above cannot capture. Ten candidate socioeconomic covariates were initially considered: four composite indices calculated by the PLANURB from 2010 Census data (Poverty/Income (IRP), Environmental Sanitation (ISA), Housing Quality (IMO), and Education (IED))and six indicators derived directly from 2010 Census household counts: the proportion of households with bathroom access, piped sewage, piped water, and direct waste collection (each expressed as a proportion of permanent private households), and mean household income, calculated both per capita and per household.

Pairwise Pearson correlations among the ten remaining covariates were examined; several pairs exceeded r = 0.80 (e.g., income per capita and income per household, r = 0.93), consistent with expected redundancy between related socioeconomic indicators. Rather than applying an a priori exclusion threshold, all ten covariates were retained for an initial model, and variable selection was performed using the double-penalty approach implemented in the *mgcv* package [24], which shrinks the smooth term of uninformative covariates towards zero without requiring stepwise hypothesis testing.

The outcome was the annual number of CVD deaths in each of the 74 neighbourhoods over the 2013–2022 period (740 neighbourhood-year observations), modelled as:


$$\begin{array}{c} \text{Y}(\text{s},\text{t})\;\sim \text{ offset}(\text{log Pop}(\text{s},\text{t}))\;+\;\Sigma \text{ f}\_\text{k}(\text{x}\_\text{k}(\text{s},\text{t}))\;+\text{ f}(\text{ano})\;+\text{ te}(\text{x}\_\text{s},\text{ y}\_\text{s},\text{ t}) \end{array}$$

where Y(s,t) is the observed death count in neighbourhood s in year t, Pop(s,t) is the corresponding annual population denominator (Section “Data sources”), f_k(·) are penalised thin-plate regression splines (k = 5 basis functions) for each socioeconomic covariate x_k, f(ano) is a smooth function of calendar year, and te(x_s, y_s, t) is a tensor-product smooth interaction between the neighbourhood centroid coordinates and year, capturing spatially and temporally varying residual risk not explained by the socioeconomic covariates. Because the socioeconomic covariates were derived from the 2010 Census and are therefore time-invariant, their estimated associations reflect cross-sectional (between-neighbourhood) rather than within-neighbourhood temporal effects; temporal variation in mortality risk is captured separately by f(ano) and the space-time interaction term.

Both Poisson and negative binomial error distributions were evaluated; the model was fitted on data from 2013–2021 and validated by predicting neighbourhood-level counts for 2022 (the most recent year), with model choice based on the lower mean squared prediction error. The negative binomial distribution provided a marginally better fit and was adopted for the final model, fitted using restricted maximum likelihood (REML) on the complete 2013–2022 dataset. After variable selection, a final, simplified model retaining only covariates with non-zero estimated degrees of freedom was refitted and compared with the full ten-covariate model using the Akaike Information Criterion (AIC).

Model adequacy was assessed through residual diagnostics: spatial autocorrelation in Pearson residuals was evaluated separately for each study year using Global Moran’s I (999 Monte Carlo permutations, Queen contiguity, row-standardised weights, consistent with the spatial analysis described above), and temporal autocorrelation was assessed using the Durbin-Watson test applied to the annual mean residual series.

A significance level of 5% (α = 0.05) was adopted for all tests.

## Results

During 2013–2022, 59,392 deaths were registered in Campo Grande. Based on the absolute counts, 8,148 (13.7%) had a cardiac disease as the underlying cause. IHD accounted for 6,476 deaths (79.5%), followed by HF (558; 6.8%), ARR (513; 6.3%), CMP (419; 5.1%), and INF (182; 2.2%). Within IHD, acute myocardial infarction (ICD-10 code I21) was responsible for 5,569 deaths (86.0% of IHD); detailed diagnosis-level distributions are presented in Supporting Information S1 Fig, as subsequent analyses in this manuscript are reported at the disease-group level (IHD, HF, ARR, CMP, INF) rather than by individual ICD-10 diagnosis.

The median age at death was 70 years (IQR: 60–80). Age varied significantly across groups (Kruskal-Wallis p < 0.001): ARR had the highest median (79 years), followed by HF (76), CMP (71), IHD (69), and INF (63 years). Males accounted for 59.3% of deaths (n = 4,832), with predominance in IHD (61.1%), CMP (57.5%) and INF (61.5%), while ARR and HF were the groups with female predominance (52.0%; 50.9%) (p < 0.001). White individuals comprised 54.6% of deaths (n = 4,452), followed by Mixed (40.0%; n = 3,260). Primary education was the most frequent level (47.8%; n = 3,893). Overall, 40.7% of deaths occurred in hospitals (n = 3,317) and 38.9% at home (n = 3,116). A distinctive pattern emerged for IHD: home deaths (43.9%; n = 2,843) surpassed hospital deaths (32.7%; n = 2,119), contrasting sharply with other groups where hospital deaths predominated (59.4–89.9%). Further details are presented in Table 1.

### Temporal trends

Annual crude and age-standardised mortality rates (per 100,000 population) by cardiovascular disease group are presented in Fig 1, Supporting Information S2 Table, and Supporting Information S2 Fig. Age-standardised rates tracked closely with crude rates (Supporting Information S2 Fig), confirming that the observed increase was not driven solely by population ageing, and IHD was consistently the leading cause of CVD death throughout the study period.

**Fig 1:**
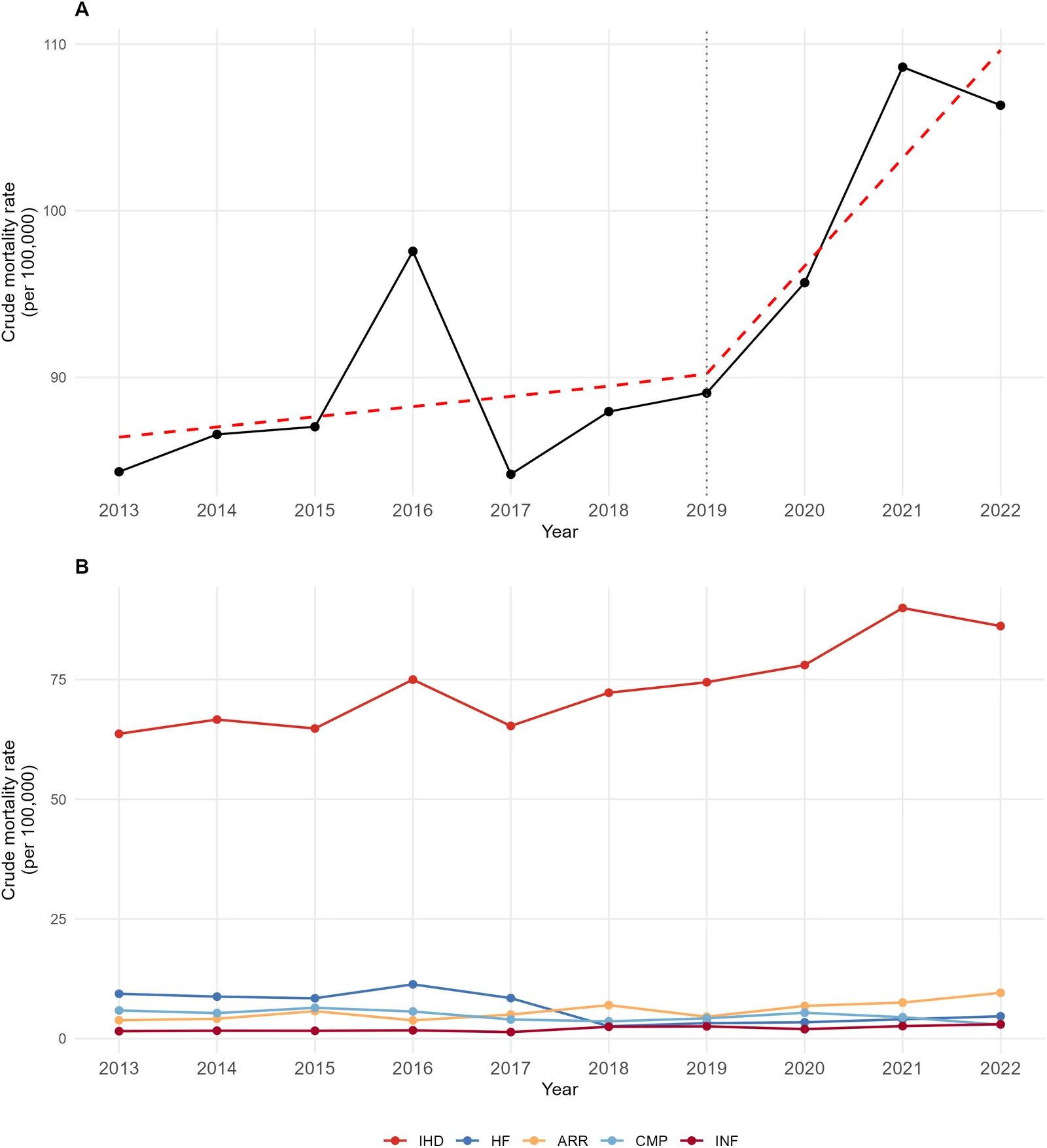
Temporal trends in cardiovascular mortality rates in Campo Grande, Mato Grosso do Sul, Brazil, 2013–2022. (A) Overall crude mortality rate (solid line) with joinpoint regression fit (dashed red line). The dotted vertical line indicates the identified breakpoint at 2019. (B) Disease-specific crude mortality rates over time. IHD: ischaemic heart disease; HF: heart failure; ARR: arrhythmias; CMP: cardiomyopathies; INF: inflammatory heart diseases.

The overall crude mortality rate increased from 84.3 per 100,000 in 2013 to 106.3 per 100,000 in 2022, a 26.1% increase. Joinpoint analysis identified a single breakpoint at 2019 (Fig 1A): before this point, the trend was non-significant (slope: + 0.6/100,000 per year); after it, a significant acceleration was observed (slope: + 6.5/100,000 per year). Poisson regression yielded an overall APC of +2.5% per year (95% CI: 1.7–3.3; p < 0.001). Overdispersion was detected in the group-specific Poisson models for IHD, HF, and ARR; negative binomial regression yielded similar APC estimates (3.65%, −11.81%, and 9.28%, respectively), confirming the robustness of the findings.

Group-specific trends showed marked heterogeneity (Table 2, Fig 1B): IHD increased at +3.7% per year (95% CI: 2.8–4.6), ARR at +9.4% (95% CI: 6.1–12.9), and INF at +7.7% (95% CI: 2.3–13.5), while HF declined at −11.9% per year (95% CI: −14.5 to −9.3) and CMP at −5.3% (95% CI: −8.4 to −2.0; all p < 0.05). Prais-Winsten regression confirmed increasing trends for IHD (β = 2.66; p < 0.001), ARR (β = 0.49; p < 0.001), and INF (β = 0.15; p = 0.002); notably, in contrast to the earlier (pre-correction) analysis, Prais-Winsten also identified statistically significant decreasing trends for HF (β = −0.73; p = 0.037) and CMP (β = −0.27; p = 0.036), reclassifying both groups from stationary to decreasing (Table 2).

**Table 2:** Annual percentage change (Poisson regression) and Prais-Winsten regression for cardiovascular mortality by disease group.

Group	APC % (95% CI)	p (Poisson)	Beta PW (SE)	p (PW)	Trend
IHD	3.70 (2.81 to 4.59)	<0.001	2.663 (0.441)	<0.001	Increasing
HF	−11.92 (−14.53 to −9.26)	<0.001	−0.730 (0.292)	0.037	Decreasing
ARR	9.43 (6.11 to 12.90)	<0.001	0.494 (0.097)	<0.001	Increasing
CMP	−5.27 (−8.41 to −2.04)	0.002	−0.269 (0.106)	0.036	Decreasing
INF	7.73 (2.34 to 13.49)	0.005	0.151 (0.032)	0.002	Increasing

APC: annual percentage change from Poisson regression with population offset. Overdispersion was detected for IHD, HF, and ARR (deviance/df ratio: 2.24, 6.77, and 1.72, respectively); negative binomial regression confirmed similar estimates (APC: 3.65%, −11.81%, and 9.28%, respectively). β PW: slope coefficient from Prais-Winsten regression (change in rate per 100,000 per year). SE: standard error.

### Sex-specific analysis and age distribution

Males consistently exhibited higher CVD mortality rates than females throughout the study period. The proportional distribution of deaths by sex across CVD groups is shown in Supporting Information S3A Fig: the male-to-female ratio varied markedly, with INF showing the greatest male excess (1.60), followed by IHD (1.57) and CMP (1.35), while HF (0.97) and ARR (0.92) showed a slight female predominance. Sex-specific mortality rates over time are presented in Supporting Information S3B Fig. The overall incidence rate ratio (IRR) was 1.59 (95% CI: 1.46–1.74), indicating 59% higher mortality in men. The sex-by-year interaction was not significant (p = 0.47), suggesting parallel temporal trends between sexes.

Elderly individuals (≥60 years) accounted for 74.9% of deaths (n = 6,100). The age–sex pyramid (Supporting Information S4 Fig) illustrates the concentration of CVD mortality in the 60–69, 70–79, and ≥80 age groups, with male predominance across most age strata.

### Spatial analysis

Choropleth maps of neighbourhood-level CVD mortality rates (Fig 2) revealed marked spatial heterogeneity across all disease groups, comparing crude and BYM-smoothed rates side by side. IHD displayed the highest rates (peak: 157.1 per 100,000 crude; 145.9 per 100,000 smoothed), followed by HF (peak: 25.2 crude; 11.4 smoothed) and ARR (peak: 24.2 crude; 8.8 smoothed), with spatial distributions closely overlapping the aggregate CVD pattern, reinforcing IHD as the primary driver of cardiovascular mortality in the city. Inflammatory heart diseases exhibited the lowest rates (peak: 12.1 crude; 2.7 smoothed).

**Fig 2:**
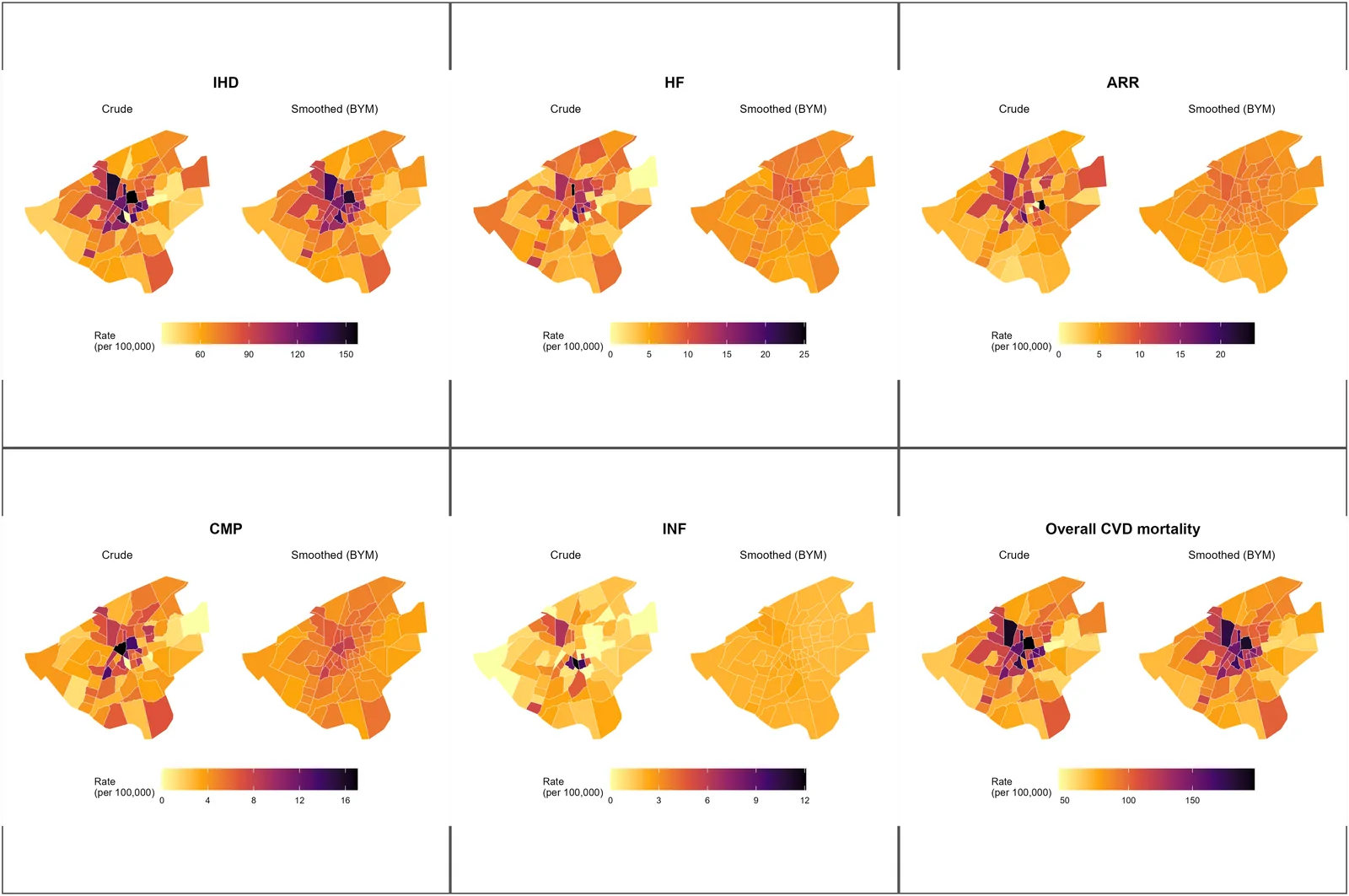
Crude and BYM-smoothed mortality rate distribution of cardiovascular disease groups by neighbourhood. Maps oriented with north at the top. Map was made in R version 4.5.1 (package *ggplot2*) using shapefiles from SIMGEO, Campo Grande City Hall (SEMADUR) [18].

KDE maps (Fig 3) identified a predominant concentration of deaths in the central districts across all groups, with an aggregate maximum density of 67.3 cases per km^2^ (IHD: 52.6; HF: 5.2; ARR: 5.1; CMP: 4.8; INF: 2.6 cases per km^2^). LISA analysis (Fig 4), applied to BYM-smoothed rates, identified significant high-high clusters in 13 neighbourhoods and low-low clusters in 5 neighbourhoods in the aggregate analysis (Global Moran’s I = 0.555, p < 0.001), with heterogeneous cluster distribution across disease groups (Moran’s I ranging from 0.533 for HF to 0.735 for INF; all p < 0.001).

**Fig 3:**
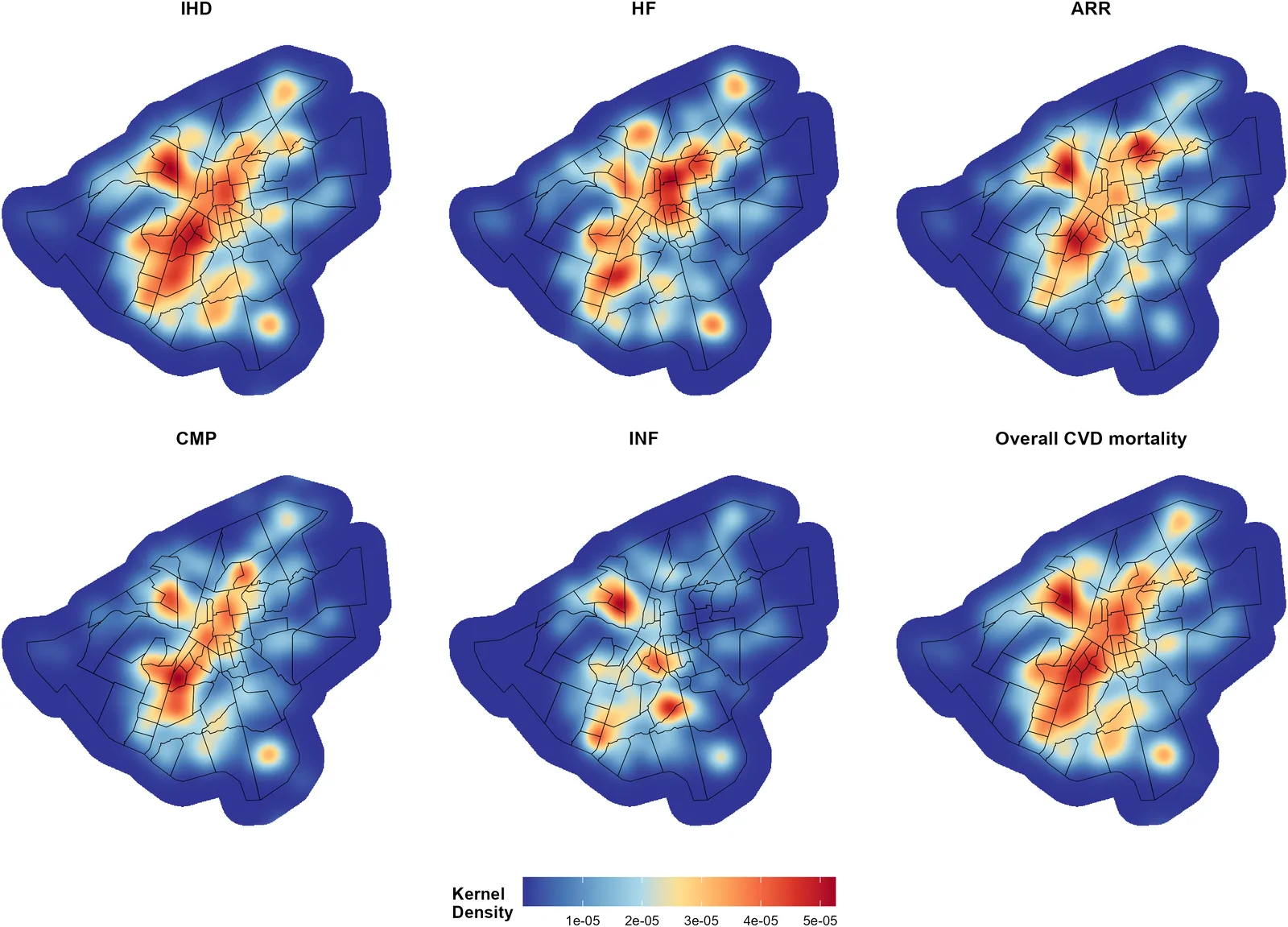
Kernel Density Estimation of deaths during the accumulated period of 2013–2022, separated by disease group. Maps oriented with north at the top. Map was made in R version 4.5.1 (package *ggplot2*) using shapefiles from SIMGEO, Campo Grande City Hall (SEMADUR) [18].

**Fig 4:**
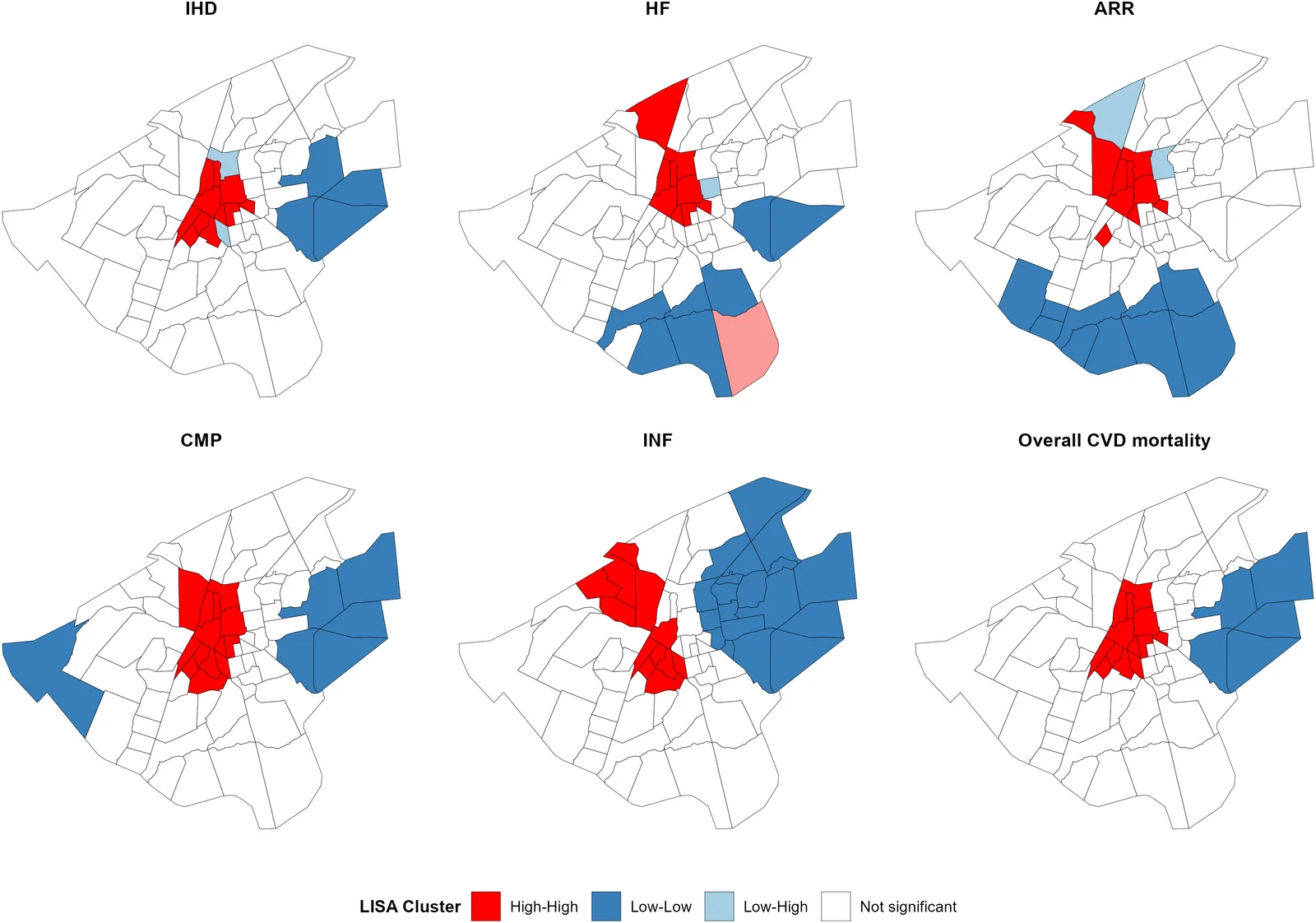
LISA cluster analysis of BYM-smoothed cardiovascular mortality rates, separated by disease group. Maps oriented with north at the top. Map was made in R version 4.5.1 (package *ggplot2*) using shapefiles from SIMGEO, Campo Grande City Hall (SEMADUR) [18].

Spearman’s correlation coefficients between BYM-smoothed neighbourhood-level mortality rates and ten socioeconomic indicators are presented in Supporting Information S5 Fig. Coefficients ranged from −0.40 to 0.63. IHD, HF, CMP, and ARR showed a consistent pattern of positive, significant correlations with socioeconomic advantage indicators (education, sanitation, income, and infrastructure access), suggesting higher mortality in more socioeconomically advantaged neighbourhoods. In contrast, the inflammatory group showed negative and significant correlations with piped water access and housing quality, indicating greater burden in more vulnerable areas.

As a complementary robustness analysis, a generalised additive model (GAM) with negative binomial errors was fitted to assess non-linear associations between neighbourhood-level CVD mortality counts and socioeconomic covariates, incorporating a space-time interaction smooth term (Table 3). Among the ten socioeconomic covariates initially considered, five were retained after penalised variable selection: the Poverty/Income Index (IRP), Housing Quality Index (IMO), proportion of households with bathroom access, proportion with piped sewage access, and mean household income. All five covariates were significantly associated with CVD mortality (p < 0.05), alongside a significant space-time interaction term (p < 0.001), indicating spatially and temporally varying mortality risk beyond that explained by the socioeconomic covariates alone. The model explained 42.0% of deviance (adjusted R^2^ = 0.815). Residual diagnostics showed no significant spatial autocorrelation in any study year (Moran’s I, all p > 0.05) and no significant temporal autocorrelation (Durbin-Watson = 2.74, p = 0.81), indicating that the model adequately captured the spatial and temporal structure of the data.

**Table 3:** Generalised additive model (GAM) of neighbourhood-level cardiovascular disease mortality counts, showing estimated degrees of freedom (edf) and significance of smooth terms for retained socioeconomic covariates, calendar year, and the space–time interaction.

Covariate	edf	Chi-sq	p-value
Poverty/Income Index	3.29	33.32	<0.001
Housing Quality Index	2.31	30.41	<0.001
Bathroom Acess	1.65	5.92	0.014
Sewage Acess	2.03	25.13	<0.001
Income per household	2.53	12.84	<0.001
Year	2.27	9.59	0.003
Space-time interaction	19.39	94.24	<0.001

Model: negative binomial GAM with log link, offset by the natural logarithm of the annual neighbourhood population (θ = 84.6); n = 740 neighbourhood-year observations (74 neighbourhoods × 10 years). edf: estimated degrees of freedom of the penalised smooth term (edf = 1 indicates a linear effect; edf > 1 indicates non-linearity). Chi-sq: approximate chi-squared test statistic for the smooth term. Space–time interaction: tensor-product smooth of neighbourhood centroid coordinates and year. Adjusted R^2^ = 0.815; deviance explained = 42.0%.

## Discussion

This study examined the epidemiological profile, temporal trends, spatial distribution, and socioeconomic correlates of CVD mortality in Campo Grande over a ten-year period, representing the first population-based analysis of this kind in a municipality of the Central-West region of Brazil. Three findings stand out for their public health relevance: the marked acceleration of CVD mortality after 2019, the high burden of out-of-hospital ischaemic deaths, and the spatial concentration of risk in identifiable, socioeconomically distinct neighbourhoods. Together, these findings indicate that cardiovascular prevention in Campo Grande, and potentially in other medium-sized Brazilian cities, would benefit from geographically and temporally targeted strategies rather than uniform, city-wide interventions.

IHD constituted nearly four-fifths of CVD deaths, consistent with national data [25,26]. Its increasing trend (+3.7% per year) contrasts with the national decline in age-standardised rates [5,6], reflecting the geographically uneven nature of this improvement, with the Central-West region showing slower progress [7,27].

Several regional factors may help explain this divergence from the national trend. First, the epidemiological transition in the Central-West region has historically lagged behind the more urbanised South and Southeast, with slower gains in risk factor control and later expansion of specialised cardiovascular care networks [7,27]. Second, population ageing, a major driver of absolute IHD burden, has proceeded unevenly across Brazilian regions, and Campo Grande’s demographic profile, shaped by substantial migration inflows as a border-state capital, may differ from the national average in ways not fully captured by crude comparisons. Third, national geospatial analyses have consistently shown that cardiac diagnostic testing and percutaneous coronary intervention services are concentrated in large urban centres in the more economically developed South and Southeast, with comparatively reduced density of high-complexity cardiac care in the Central-West, North, and Northeast regions [28]. Consistent with this pattern, greater geographic distance from interventional cardiology centres has been independently associated with higher ischaemic heart disease mortality in another Brazilian state, even after adjusting for socioeconomic factors [29].

A particularly significant finding was the high proportion of IHD deaths at home (43.9%), surpassing hospital deaths (32.7%) and contrasting sharply with other CVD groups (59–90% hospital deaths). Similar patterns have been reported for AMI in Brazilian state capitals [30], and likely reflect delayed symptom recognition and access barriers, underscoring the need for community education campaigns, strengthened emergency response capacity, and wider distribution of automated external defibrillators.

Beyond individual-level factors, the high proportion of out-of-hospital IHD deaths observed in this study may also reflect structural characteristics of the local emergency care network. Prehospital delay for acute coronary syndrome in Brazil is substantially longer than international benchmarks, driven predominantly by system-level factors, including limited emergency medical service utilisation, rather than patient-related delay alone; a Brazilian single-centre study reported a median total reperfusion time of 430 minutes, with system delay accounting for 81.1% of this interval and emergency medical services used in only 23.5% of cases [31]. Nationally, patients relying on the public health system have been shown to experience markedly longer prehospital delays and lower rates of timely reperfusion than those treated in the private sector [32]. Regarding the organisation and availability of emergency services, a national ecological study found that over 14 million Brazilians live 120 km or more from a high-complexity emergency facility with adult intensive care capacity [33], and a geospatial analysis of emergency care sensitive conditions identified that nearly 70% of high-burden municipal hotspots were more than 180 minutes’ driving distance from the nearest emergency facility [34]. Telemedicine-supported prehospital networks, including remote electrocardiogram interpretation and cardiology teleconsultation, have shown promise in mitigating distance-related delay and improving outcomes in acute coronary syndrome in Brazil [35], and may represent a relevant strategy for a geographically extensive, comparatively low-density state such as Mato Grosso do Sul. Primary Health Care also plays a central role in the longitudinal identification and management of cardiovascular risk factors before an acute event occurs, including through programmes such as HIPERDIA for the registration and monitoring of patients with hypertension and diabetes mellitus [9]; strengthening this preventive function, alongside emergency network capacity, may be necessary to reduce the burden of out-of-hospital IHD mortality in Campo Grande.

The declining trend in HF mortality aligns with national findings [36,37], with classification by Prais-Winsten regress method. The marked increase in ARR mortality may partly reflect improvements in diagnostic and coding practices [3], alongside population ageing and rising atrial fibrillation prevalence, consistent with the observed female predominance in this group. Male predominance in overall CVD mortality (IRR = 1.59) is well-established in the literature [38,39], and the stable sex gap over time suggests these determinants have not shifted substantially in Campo Grande. The concentration of deaths among individuals with primary education mirrors national data linking lower educational attainment to higher cardiovascular mortality [40,41]. The acceleration in CVD mortality after 2019 is consistent with COVID-19’s documented impact, including excess cardiovascular deaths, reduced hospitalisations, and direct myocardial effects of SARS-CoV-2 [42–44]. However, this study’s ecological, observational design does not permit direct attribution of this acceleration to COVID-19, and this temporal association should be interpreted as hypothesis-generating rather than causal. This finding should be interpreted with caution, as the observed increase may partly reflect attribution bias: deaths directly caused by COVID-19 may have been coded as IHD or other cardiovascular causes, particularly in the early pandemic period when testing capacity was limited and COVID-19 underreporting was substantial in Brazil [44]. This misattribution would artificially inflate CVD mortality counts during 2020–2022, making it difficult to disentangle true increases in cardiovascular burden from coding artefacts. Future studies using multiple-cause-of-death analysis would be better positioned to quantify the independent contribution of COVID-19 to the observed acceleration.

Spatial analysis revealed marked neighbourhood-level heterogeneity across disease groups. IHD and HF exhibited the highest mortality rates, with spatial distributions closely overlapping the aggregate CVD pattern, reinforcing IHD as the primary driver of cardiovascular burden. KDE identified a predominant concentration of deaths in central districts, while LISA identified significant high-high clusters in the central area for IHD, with remaining groups, although having central area distribution, show a more diffuse distribution associated with peripheral, socioeconomically vulnerable areas, underscoring the need for geographically targeted interventions. The substantially higher number of significant spatial clusters identified using BYM-smoothed rates compared to crude rates reflects the increased statistical power of Bayesian smoothing to detect true underlying spatial patterns by reducing the instability associated with small case counts in low-population neighbourhoods, a well-documented property of this method. The socioeconomic analysis revealed heterogeneous associations. IHD, CMP and ARR showed significant correlations with neighbourhood-level indicators, suggesting higher mortality in more socioeconomically advantaged neighbourhoods. HF showed weak-to-moderate positive correlations with urban quality of life, education, sanitation, and income indicators. Three complementary mechanisms may explain this pattern: first, HF is a chronic, age-related condition that disproportionately manifests in longer-lived populations concentrated in more developed areas [25,45]; second, differential survival may operate whereby residents of socioeconomically vulnerable areas die earlier from competing causes and do not reach the age at which HF emerges as the underlying cause of death; third, unequal access to diagnosis may introduce classification bias, as deaths in vulnerable areas may be registered as non-specific cardiac conditions or IHD rather than HF, artificially reducing HF rates in peripheral neighbourhoods [37,45]. Inflammatory diseases showed the opposite pattern, with negative correlations indicating greater burden in socioeconomically vulnerable areas, consistent with their infectious aetiology and association with inadequate sanitation.

This study’s strengths include decade-long coverage, three complementary methods for temporal analysis, pathophysiologically meaningful disease disaggregation, and integration of spatial and socioeconomic analyses at the neighbourhood level. Several limitations should be acknowledged. The ecological design precludes individual-level causal inference and is subject to the ecological fallacy, whereby associations observed at the neighbourhood level do not necessarily reflect relationships that would be observed at the individual level; results may additionally be sensitive to the choice of spatial unit (modifiable areal unit problem). Misclassification of underlying cause of death is a recognised limitation of mortality data systems, and attribution bias may have affected cardiovascular coding during the pandemic period. Intercensal population estimates introduce uncertainty in rate calculations, particularly for smaller neighbourhoods. Neighbourhood-level mortality rates were not age-standardised, owing to the unavailability of age-stratified population data at this spatial resolution; consequently, differences in age structure across neighbourhoods may partly explain the observed spatial heterogeneity in crude and BYM-smoothed rates. Socioeconomic indicators were derived from the 2010 Census, introducing temporal misalignment with the study period; although structural neighbourhood characteristics change slowly, significant urban transformations may not be fully captured. Administrative data censoring and positional uncertainty inherent to the geocoding process may also introduce residual misclassification in spatial analyses; additionally, 69 cases (0.85% of confirmed residents) could not be assigned to a neighbourhood, owing to rural residence or unresolved address matching, and were excluded from the neighbourhood-level spatial analysis, although retained in all non-spatial analyses. Finally, the inability to assess individual-level risk factors, including smoking, hypertension, diabetes, and dyslipidaemia, further limits causal inference.

## Conclusion

Cardiovascular mortality in Campo Grande increased significantly between 2013 and 2022, driven primarily by ischaemic heart disease, with heterogeneous trends across disease groups: arrhythmia and inflammatory heart disease mortality increased, while heart failure and cardiomyopathy mortality declined significantly, a finding confirmed, rather than reclassified as non-significant, after correction for serial autocorrelation. Spatial analysis revealed concentration of deaths in central districts, significant High-High clusters, and marked variation in socioeconomic correlates across groups. The high proportion of out-of-hospital IHD deaths underscores the need to strengthen primary health care and optimise emergency care network response capacity, alongside localised interventions targeting socioeconomically vulnerable neighbourhoods. As the first population-based study of CVD mortality at the municipal level in the Central-West region, these findings support locally tailored cardiovascular prevention strategies in medium-sized Brazilian cities. Health authorities may use this study to strengthen cardiovascular epidemiological surveillance and integrate it with Primary Health Care. Additionally, areas with the highest mortality burden may be prioritised for targeted prevention and early diagnosis strategies. Future research should investigate the role of geographic distance and pre-hospital response time on avoidable cardiovascular deaths.

## Supporting information

S1 Table Geocoding precision of cardiovascular deaths among confirmed Campo Grande residents, 2013–2022 (N = 8,148). (PDF)

S2 Table Crude and age-standardised mortality rates (per 100,000 population) by cardiovascular disease group, Campo Grande, MS, 2013–2022. (PDF)

S1 Fig Distribution of cardiovascular disease mortality by group and ICD-10 diagnoses. (PDF)

S2 Fig Comparison of crude and WHO age-standardised overall cardiovascular mortality rates, Campo Grande, MS, 2013–202. (PDF)

S3 Fig Cardiovascular mortality by sex in Campo Grande, Mato Grosso do Sul, Brazil, 2013–2022. (PDF)

S4 Fig Age–sex pyramid of cardiovascular deaths in Campo Grande, Mato Grosso do Sul, Brazil, 2013–2022. (PDF)

S5 Fig Spearman’s rank correlation coefficient separated by group. (PDF)
