# Supplementary material for: Cardiovascular disease mortality in a Central-West Brazilian capital: A spatial, temporal, and ecological analysis
Source: PLoS One. 2026 Sep 29;21(9):e0359337. doi: 10.1371/journal.pone.0359337 (PMC13623126; doi:10.1371/journal.pone.0359337)
Supplement: Supplementary file 1 [file pone.0359337.s001.pdf]

Cardiovascular disease mortality in a Central-West Brazilian capital: a spatial, temporal, and ecological analysis

João Vitor Barrio, Rafael Santana Batista Cabral, Sonia Regina Maciel Rodrigues da Silva, Audêncio Victor, Maria Elizabeth Araújo Ajalla, Cláudia Du Bocage Santos-Pinto, Everton Falcão de Oliveira

Supporting Information Table S1. Geocoding precision of cardiovascular deaths among confirmed Campo Grande residents, 2013–2022 (N = 8,148)

**Match precision**	**n**	**%**
Exact street address	7,469	91.7
Street-level interpolation	257	3.2
Neighbourhood/city centroid	184	2.3
Postal code (CEP)	172	2.1
Region/state level	65	0.8
Sub-address	1	0.01
**Total**	**8,148**	**100.0**
