# Supplementary material for: Cardiovascular disease mortality in a Central-West Brazilian capital: A spatial, temporal, and ecological analysis
Source: PLoS One. 2026 Sep 29;21(9):e0359337. doi: 10.1371/journal.pone.0359337 (PMC13623126; doi:10.1371/journal.pone.0359337)
Supplement: Supplementary file 2 [file pone.0359337.s002.pdf]

Cardiovascular disease mortality in a Central-West Brazilian capital: a spatial, temporal, and ecological analysis

João Vitor Barrio, Rafael Santana Batista Cabral, Sonia Regina Maciel Rodrigues da Silva, Audêncio Victor, Maria Elizabeth Araújo Ajalla, Cláudia Du Bocage Santos-Pinto, Everton Falcão de Oliveira

Supporting Information Table S2. Crude and age-standardised mortality rates (per 100,000 population) by cardiovascular disease group, Campo Grande, MS, 2013–2022

Year	IHD Crude rate (age-standardised rate)	HF Crude rate (age-standardised rate)	ARR Crude rate (age-standardised rate)	CMP Crude rate (age-standardised rate)	INF Crude rate (age-standardised rate)
2013	63.7 (117.2)	9.4 (18.8)	3.8 (8.0)	5.9 (10.6)	1.5 (10.6)
2014	66.6 (119.9)	8.8 (17.3)	4.1 (8.7)	5.3 (9.9)	1.6 (3.2)
2015	64.7 (119.4)	8.4 (18.7)	5.7 (13.5)	6.4 (12.7)	1.6 (2.3)
2016	75.0 (142.6)	11.3 (26.0)	3.8 (8.6)	5.7 (11.3)	1.7 (2.3)
2017	65.3 (120.5)	8.4 (16.9)	5.0 (11.2)	4.0 (8.0)	1.4 (1.9)
2018	72.2 (132.7)	2.6 (5.4)	7.0 (14.7)	3.6 (6.8)	2.5 (4.0)
2019	74.4 (142.0)	3.2 (7.0)	4.6 (10.0)	4.2 (7.6)	2.5 (3.8)
2020	78.0 (142.0)	3.4 (7.2)	6.9 (15.0)	5.4 (9.2)	89.9 (168.6)
2021	89.9 (168.6)	4.0 (7.3)	7.5 (16.6)	4.5 (8.8)	2.6 (4.2)
2022	86.2 (162.9)	4.7 (8.9)	9.6 (21.1)	2.9 (5.9)	3.0 (4.6)

IHD: ischaemic heart disease; HF: heart failure; ARR: arrhythmias; CMP: cardiomyopathies; INF: inflammatory heart diseases.
