# Supplementary material for: Cardiovascular disease mortality in a Central-West Brazilian capital: A spatial, temporal, and ecological analysis
Source: PLoS One. 2026 Sep 29;21(9):e0359337. doi: 10.1371/journal.pone.0359337 (PMC13623126; doi:10.1371/journal.pone.0359337)
Supplement: Supplementary file 3 [file pone.0359337.s003.pdf]

Cardiovascular disease mortality in a Central-West Brazilian capital: a spatial, temporal, and ecological analysis

João Vitor Barrio, Rafael Santana Batista Cabral, Sonia Regina Maciel Rodrigues da Silva, Audêncio Victor, Maria Elizabeth Araújo Ajalla, Cláudia Du Bocage Santos-Pinto, Everton Falcão de Oliveira

Supporting Information Fig S1. Distribution of cardiovascular disease mortality by group and ICD-10 diagnoses

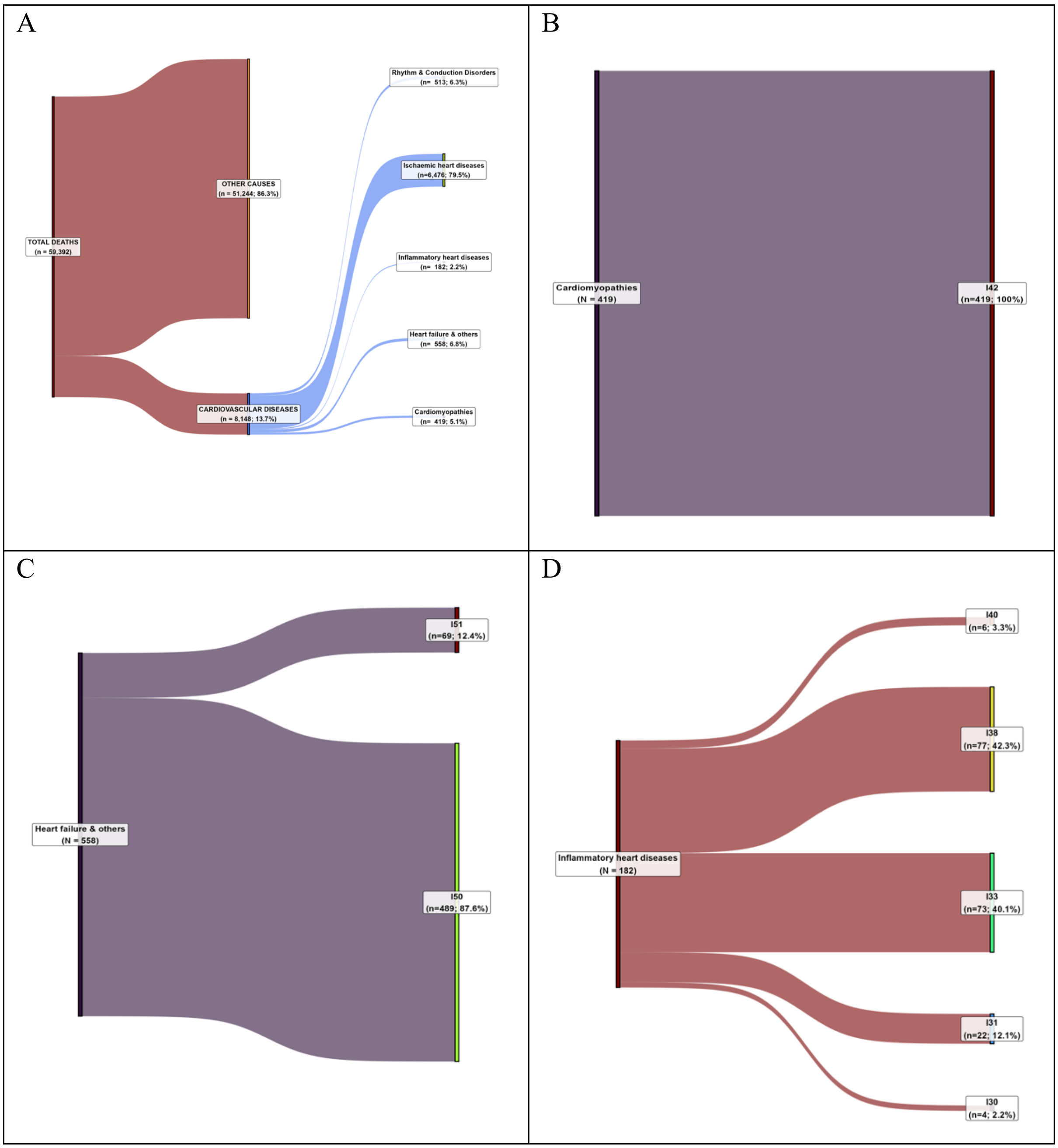

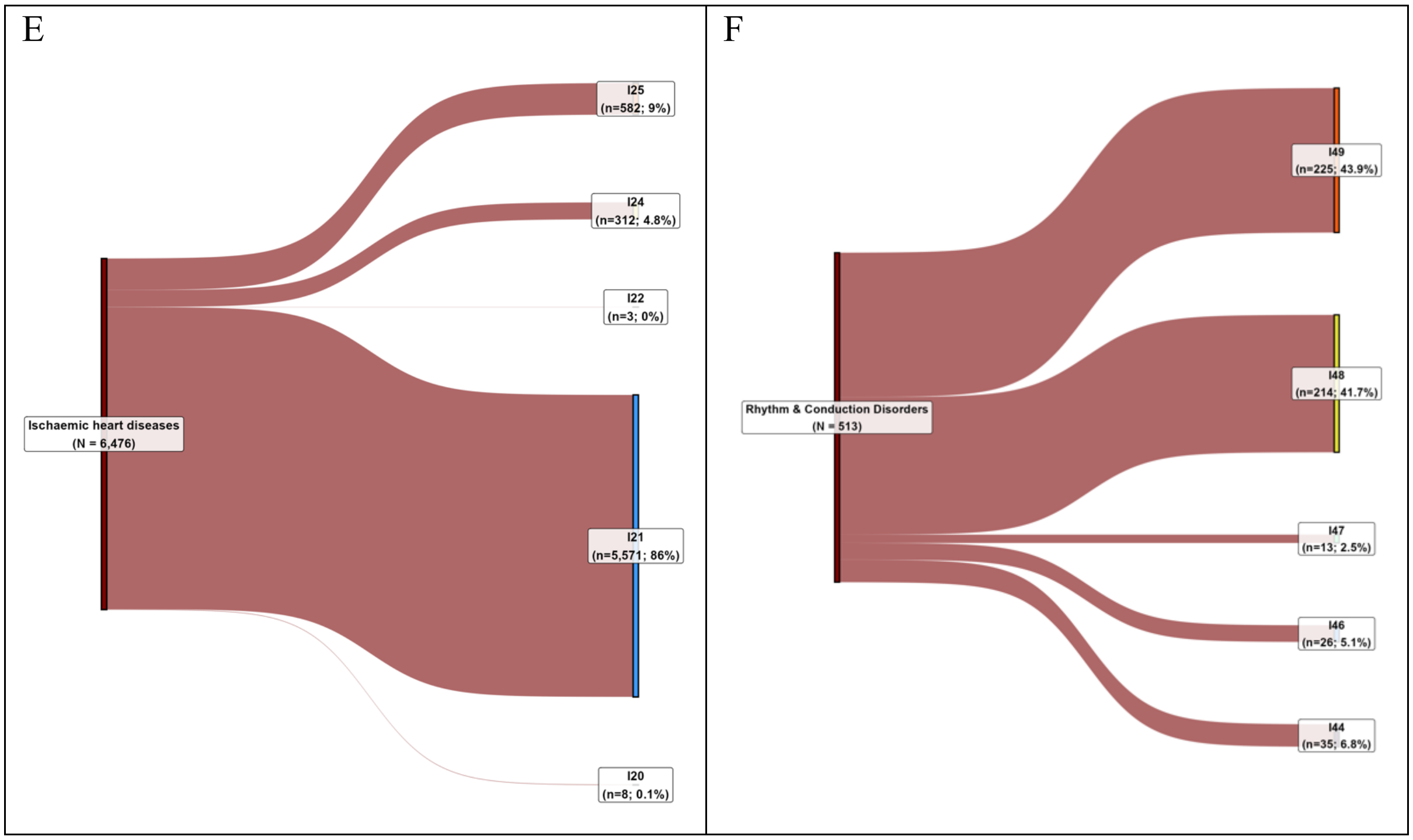

The Sankey plots illustrate the flow and proportion of deaths, based on the five groups and their ICD-10 distribution. (A) Magnitude of Cardiovascular Diseases compared to total deaths in the study period. (B) Cardiomyopathies (C) Heart Failure and others (D) Inflammatory heart diseases (E) Ischemic Heart Diseases. (F) Rhythm and conduction disorders. ICD-10: International Classification of Diseases, 10th Revision.
