# Supplementary material for: Cardiovascular disease mortality in a Central-West Brazilian capital: A spatial, temporal, and ecological analysis
Source: PLoS One. 2026 Sep 29;21(9):e0359337. doi: 10.1371/journal.pone.0359337 (PMC13623126; doi:10.1371/journal.pone.0359337)
Supplement: Supplementary file 5 [file pone.0359337.s005.pdf]

Cardiovascular disease mortality in a Central-West Brazilian capital: a spatial, temporal, and ecological analysis

João Vitor Barrio, Rafael Santana Batista Cabral, Sonia Regina Maciel Rodrigues da Silva, Audêncio Victor, Maria Elizabeth Araújo Ajalla, Cláudia Du Bocage Santos-Pinto, Everton Falcão de Oliveira

Supporting Information Fig S3. Cardiovascular mortality by sex in Campo Grande, Mato Grosso do Sul, Brazil, 2013–2022

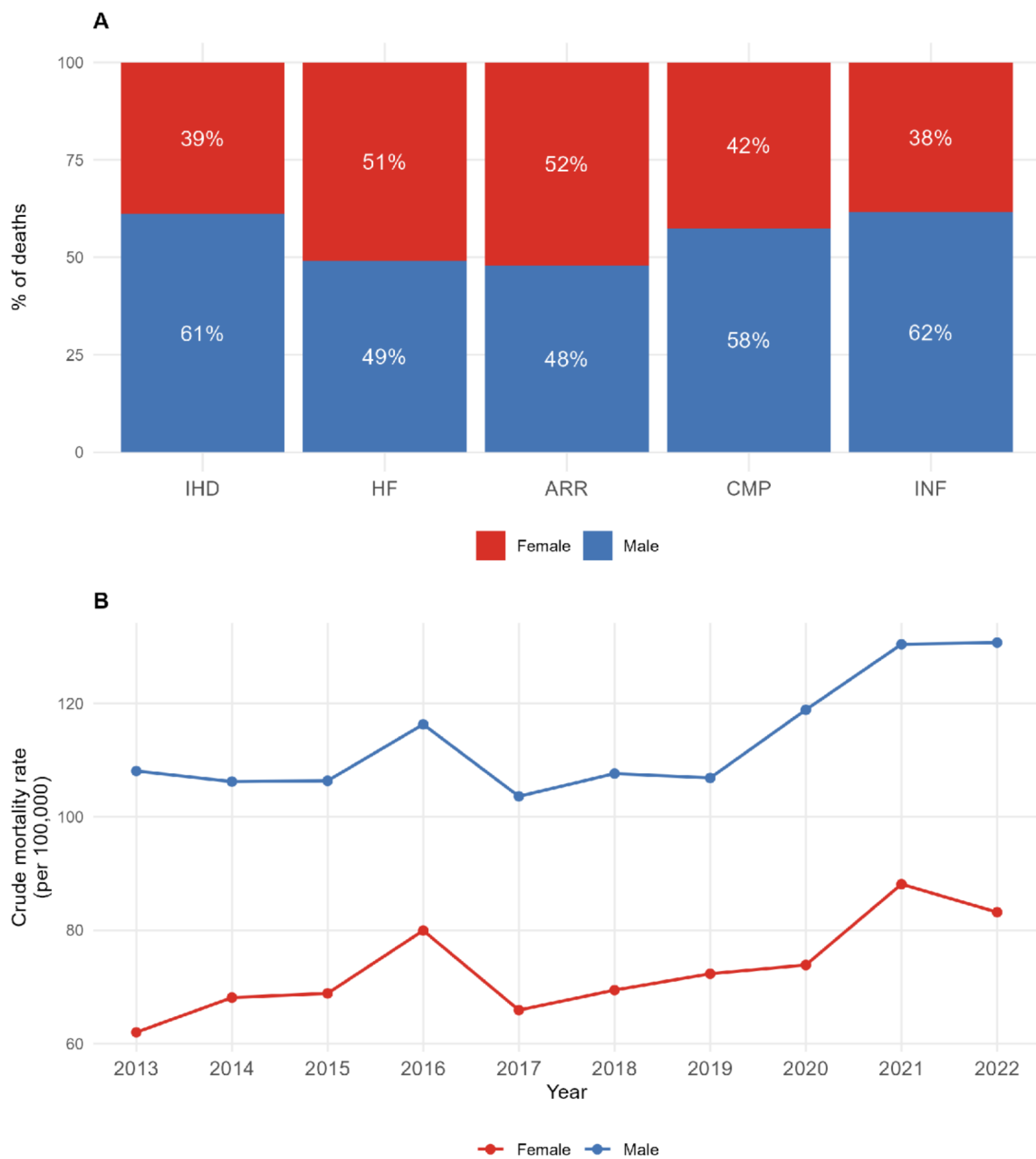

(A) Proportional distribution of deaths by sex across cardiovascular disease groups. Percentages shown above each bar. (B) Sex-specific crude mortality rates over time (per 100,000 population). IRR (male vs female) = 1.59 (95% CI: 1.46–1.74).
