# Supplementary material for: Cardiovascular disease mortality in a Central-West Brazilian capital: A spatial, temporal, and ecological analysis
Source: PLoS One. 2026 Sep 29;21(9):e0359337. doi: 10.1371/journal.pone.0359337 (PMC13623126; doi:10.1371/journal.pone.0359337)

Cardiovascular disease mortality in a Central-West Brazilian capital: a spatial, temporal, and ecological analysis

João Vitor Barrio, Rafael Santana Batista Cabral, Sonia Regina Maciel Rodrigues da Silva, Audêncio Victor, Maria Elizabeth Araújo Ajalla, Cláudia Du Bocage Santos-Pinto, Everton Falcão de Oliveira

Supporting Information Fig S5. Spearman's rank correlation coefficient separated by group

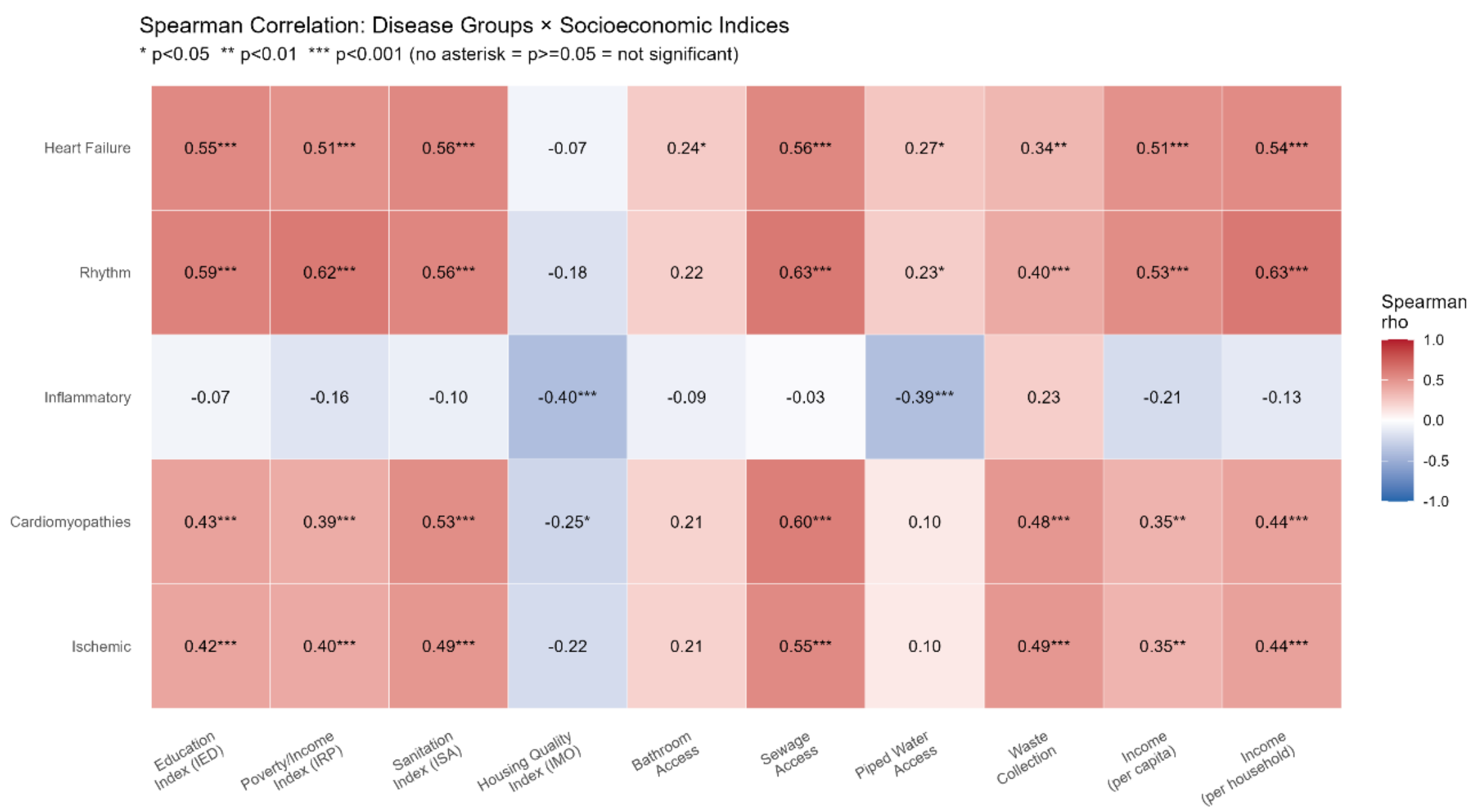

Supplement: Supplementary file 7 [file pone.0359337.s007.pdf]
