# Supplementary material for: Cardiovascular disease mortality in a Central-West Brazilian capital: A spatial, temporal, and ecological analysis
Source: PLoS One. 2026 Sep 29;21(9):e0359337. doi: 10.1371/journal.pone.0359337 (PMC13623126; doi:10.1371/journal.pone.0359337)
Supplement: Peer review history

# Peer review: Decision Letter 0

**DOI:** 10.1371/journal.pone.0359337.r001

**Authors:** Patrick R Stephens

15 Jul 2026

Dear Dr. Falcão de Oliveira,

Thank you for submitting your manuscript to PLOS ONE. After careful consideration, we feel that it has merit but does not fully meet PLOS ONE’s publication criteria as it currently stands. Therefore, we invite you to submit a revised version of the manuscript that addresses the points raised during the review process.

All of the reviewers found the study appropriate for PLOS One, and of general interest.  However, two of the reviewers identified a number of issues with the text that will need to be clarified before it will be ready for publication.  Since no new analyses or re-analyses are needed, it should be a relatively straightforward revision.

Please submit your revised manuscript by Aug 14 2026 11:59PM. If you will need more time than this to complete your revisions, please reply to this message or contact the journal office at plosone@plos.org. When you're ready to submit your revision, log on to https://www.editorialmanager.com/pone/ and select the 'Submissions Needing Revision' folder to locate your manuscript file.

- A letter that responds to each point raised by the academic editor and reviewer(s). You should upload this letter as a separate file labeled 'Response to Reviewers'.
- A marked-up copy of your manuscript that highlights changes made to the original version. You should upload this as a separate file labeled 'Revised Manuscript with Track Changes'.
- An unmarked version of your revised paper without tracked changes. You should upload this as a separate file labeled 'Manuscript'.

If you would like to make changes to your financial disclosure, please include your updated statement in your cover letter. Guidelines for resubmitting your figure files are available below the reviewer comments at the end of this letter.

If applicable, we recommend that you deposit your laboratory protocols in protocols.io to enhance the reproducibility of your results. Protocols.io assigns your protocol its own identifier (DOI) so that it can be cited independently in the future. For instructions see: https://journals.plos.org/plosone/s/submission-guidelines#loc-laboratory-protocols. Additionally, PLOS ONE offers an option for publishing peer-reviewed Lab Protocol articles, which describe protocols hosted on protocols.io. Read more information on sharing protocols at https://plos.org/protocols?utm_medium=editorial-email&utm_source=authorletters&utm_campaign=protocols.

As the corresponding author, your ORCID iD is verified in the submission system and will appear in the published article. PLOS supports the use of ORCID, and we encourage all coauthors to register for an ORCID iD and use it as well. Please encourage your coauthors to verify their ORCID iD within the submission system before final acceptance, as unverified ORCID iDs will not appear in the published article. *Only*  the individual author can complete the verification step; PLOS staff *cannot*  verify ORCID iDs on behalf of authors.

We look forward to receiving your revised manuscript.

Kind regards,

Patrick R Stephens, Ph.D.

Academic Editor

PLOS One

Journal Requirements:

When submitting your revision, we need you to address these additional requirements.

1. Please ensure that your manuscript meets PLOS ONE's style requirements, including those for file naming. The PLOS ONE style templates can be found at

https://journals.plos.org/plosone/s/file?id=wjVg/PLOSOne_formatting_sample_main_body.pdf and https://journals.plos.org/plosone/s/file?id=ba62/PLOSOne_formatting_sample_title_authors_affiliations.pdf

2. You have indicated that data is available from [cepconep.propp@ufms.br.]. Please can we ask you to provide us with a general contact email address for the data requests, so readers can request access in perpetuity. If a general email is not available please provide a link to a website where readers can obtain access to data.

3. Please provide a complete Data Availability Statement in the submission form, ensuring you include all necessary access information or a reason for why you are unable to make your data freely accessible. If your research concerns only data provided within your submission, please write "All data are in the manuscript and/or supporting information files" as your Data Availability Statement.

4. We note that Figure(s) 2, 3 in your submission contain [map/satellite] images which may be copyrighted. All PLOS content is published under the Creative Commons Attribution License (CC BY 4.0), which means that the manuscript, images, and Supporting Information files will be freely available online, and any third party is permitted to access, download, copy, distribute, and use these materials in any way, even commercially, with proper attribution. For these reasons, we cannot publish previously copyrighted maps or satellite images created using proprietary data, such as Google software (Google Maps, Street View, and Earth). For more information, see our copyright guidelines: http://journals.plos.org/plosone/s/licenses-and-copyright.

We require you to either (1) present written permission from the copyright holder to publish these figures specifically under the CC BY 4.0 license, or (2) remove the figures from your submission:

a. You may seek permission from the original copyright holder of Figure(s) 2, 3 to publish the content specifically under the CC BY 4.0 license.

We recommend that you contact the original copyright holder with the Content Permission Form (http://journals.plos.org/plosone/s/file?id=7c09/content-permission-form.pdf) and the following text:

“I request permission for the open-access journal PLOS ONE to publish XXX under the Creative Commons Attribution License (CCAL) CC BY 4.0 (http://creativecommons.org/licenses/by/4.0/). Please be aware that this license allows unrestricted use and distribution, even commercially, by third parties. Please reply and provide explicit written permission to publish XXX under a CC BY license and complete the attached form.”

Please upload the completed Content Permission Form or other proof of granted permissions as an "Other" file with your submission.

In the figure caption of the copyrighted figure, please include the following text: “Reprinted from [ref] under a CC BY license, with permission from [name of publisher], original copyright [original copyright year].”

b. If you are unable to obtain permission from the original copyright holder to publish these figures under the CC BY 4.0 license or if the copyright holder’s requirements are incompatible with the CC BY 4.0 license, please either i) remove the figure or ii) supply a replacement figure that complies with the CC BY 4.0 license. Please check copyright information on all replacement figures and update the figure caption with source information. If applicable, please specify in the figure caption text when a figure is similar but not identical to the original image and is therefore for illustrative purposes only.

The following resources for replacing copyrighted map figures may be helpful:

USGS National Map Viewer (public domain): http://viewer.nationalmap.gov/viewer/

The Gateway to Astronaut Photography of Earth (public domain): http://eol.jsc.nasa.gov/sseop/clickmap/

Maps at the CIA (public domain): https://www.cia.gov/library/publications/the-world-factbook/index.html and https://www.cia.gov/library/publications/cia-maps-publications/index.html

NASA Earth Observatory (public domain): http://earthobservatory.nasa.gov/

Landsat: http://landsat.visibleearth.nasa.gov/

USGS EROS (Earth Resources Observatory and Science (EROS) Center) (public domain): http://eros.usgs.gov/#

Natural Earth (public domain): http://www.naturalearthdata.com/

5. If the reviewer comments include a recommendation to cite specific previously published works, please review and evaluate these publications to determine whether they are relevant and should be cited. There is no requirement to cite these works unless the editor has indicated otherwise.

6. Please review your reference list to ensure that it is complete and correct. If you have cited papers that have been retracted, please include the rationale for doing so in the manuscript text, or remove these references and replace them with relevant current references. Any changes to the reference list should be mentioned in the rebuttal letter that accompanies your revised manuscript. If you need to cite a retracted article, indicate the article’s retracted status in the References list and also include a citation and full reference for the retraction notice.

[Note: HTML markup is below. Please do not edit.]

Reviewers' comments:

Reviewer's Responses to Questions

**Comments to the Author**

1. Is the manuscript technically sound, and do the data support the conclusions?

Reviewer #1: Yes

Reviewer #2: Yes

Reviewer #3: Yes

**********

2. Has the statistical analysis been performed appropriately and rigorously?

Reviewer #1: Yes

Reviewer #2: Yes

Reviewer #3: Yes

**********

3. Have the authors made all data underlying the findings in their manuscript fully available?

The PLOS Data policy

Reviewer #1: Yes

Reviewer #2: Yes

Reviewer #3: Yes

**********

4. Is the manuscript presented in an intelligible fashion and written in standard English?

Reviewer #1: Yes

Reviewer #2: Yes

Reviewer #3: Yes

**********

Reviewer #1: Introduction:

It is recommended that, at the end of the introduction, you state more clearly whether the study’s objective was achieved, in accordance with the journal’s guidelines.

Results:

It is suggested that the text clarify that information regarding acute myocardial infarction is not presented in tables and/or figures, thereby making it easier for the reader to understand.

When noting that most deaths occurred among men, it is recommended to list the diseases with the highest incidence in descending order, thereby facilitating the organization and interpretation of the results.

There was a discrepancy between the figures presented in the text and in Table 1 regarding the race/ethnicity variable. While the text indicates that individuals of mixed race accounted for 41.6% of deaths, Table 1 shows a percentage of 41.7%. This information needs to be revised.

An inconsistency was also found in the data on educational attainment. The text states that 48.8% of deaths were among those with a primary school education, while Table 1 shows a figure of 51.8%. Verify the figures and correct the discrepancy.

In Table 1, it is important to include the percentages of disease groups relative to the total number of deaths, to facilitate comparison among categories and provide a more comprehensive view of the distribution of the events analyzed.

Reviewer #2: The study is well designed, complies with the journal's guidelines, and presents novel and relevant evidence. The abstract is appropriate and clearly summarizes the objectives, methods, main findings, and conclusions of the study, accurately reflecting the content of the manuscript. The methodology is appropriate, the analyses are well conducted, and the findings make a valuable contribution to the existing literature. The results are robust and are presented clearly and objectively through well-designed figures, graphs, and tables that are easy to understand and effectively support the study's findings. I highly recommend this manuscript for publication.

Reviewer #3: Revision

Introduction

Point 1. Lines 58–59. It’s important to elaborate on the magnitude of the problem within the study setting by including specific epidemiological data on cardiovascular diseases in Campo Grande—such as mortality rates or the proportion of cardiovascular disease deaths relative to total deaths—and comparing the situation in Campo Grande with that of the Central-West region and Brazil as a whole, to demonstrate whether the indicators for the capital exceed state and national averages.

Point 2. Lines 62–64. The introduction states that Campo Grande presents a particularly relevant setting for the study due to factors such as its location, demographic flows, and urbanization; however, I suggest describing how these factors specifically influence cardiovascular disease mortality in the study area, thereby justifying this investigation.

Point 3. Line 65. In addition to the role of primary health care, it is important to include information regarding cardiovascular disease surveillance as addressed within the Unified Health System (SUS) by major Brazilian public health policies.

Methods

Point 4. Lines 99–105. In the methods section, clarify the inclusion and exclusion criteria for death records regarding the municipality of residence, incomplete records, and potential duplicates. State how deaths with ill-defined causes or records with missing information were handled.

Point 5. Lines 92–93. The 74 neighborhoods were treated as units of analysis for the ecological study; however, it is not stated whether they exhibit population stability, whether neighborhoods with small populations were aggregated, or whether the rates were calculated directly or subjected to smoothing.

Point 6. Lines 216–217. It is recommended to explicitly present the formulas used to calculate mortality rates, indicating the numerator and denominator and whether calculations were performed at the neighborhood level. Was spatial standardization of the rates applied?

Point 7. Line 225. For spatial analysis, it is important to present certain essential information, such as the address unit used and how unlocated addresses were handled.

Point 8. Lines 235–249. Specify the parameters used in the Kernel Density Estimation (KDE) analyses, as well as the criteria for the Global Moran's I and LISA analyses.

Results

Point 9. Results. It is recommended to clarify which of the presented results correspond primarily to crude or standardized rates, so as to avoid potential misinterpretations.

Point 10. Lines 266–271. Some results describe frequencies but do not report the total number of records included, which could indicate any exclusions made.

Point 11. Table 1. I suggest presenting information on missing data for the main variables analyzed—age, sex, race, education, marital status, and place of occurrence—given that these are secondary data from the Mortality Information System (SIM).

Discussion

Point 12. The first paragraph repeats the results. I recommend making the opening section of the discussion more interpretive, highlighting novel findings and their implications for public health.

Point 13. The association between increased mortality after 2019 and the COVID-19 pandemic is plausible; however, the discussion presents a temporal relationship that might imply causality. It is recommended to emphasize that the study does not allow for directly attributing the increase to the consequences of COVID-19.

Point 14. The study highlights the rise in ischemic heart disease (IHD) mortality in contrast to the national decline. It is recommended to discuss potential regional factors, such as varying epidemiological transitions, population aging, the prevalence of risk factors, and inequalities in cardiovascular care—including access to the cardiovascular disease care pathway.

Point 15. Home-based mortality due to IHD is a significant finding; however, I recommend discussing key aspects of this scenario, such as the availability of and access to emergency services, the time between symptom onset and care (response time), the organization of the emergency care network, and the role of Primary Health Care in identifying cardiovascular risk.

Point 16. It is important to strengthen the discussion regarding study limitations—potentially including the ecological fallacy—by noting that neighborhood-level associations do not necessarily reflect individual-level relationships.

Point 17. Include implications for health policies, particularly practical applications, especially given that this is a municipal-level study. I suggest addressing the following aspects: strengthening cardiovascular epidemiological surveillance, integrating surveillance with Primary Health Care, prioritizing areas with the highest mortality burden, and implementing prevention and early diagnosis strategies.

**********

what does this mean?). If published, this will include your full peer review and any attached files.

If you choose “no”, your identity will remain anonymous but your review may still be made public.

**Do you want your identity to be public for this peer review?**  For information about this choice, including consent withdrawal, please see our Privacy Policy

Reviewer #1: **Yes:**  Mirele Coelho Araujo

Reviewer #2: No

Reviewer #3: No

**********

[NOTE: If reviewer comments were submitted as an attachment file, they will be attached to this email and accessible via the submission site. Please log into your account, locate the manuscript record, and check for the action link "View Attachments". If this link does not appear, there are no attachment files.]

To ensure your figures meet our technical requirements, please review our figure guidelines: https://journals.plos.org/plosone/s/figures

You may also use PLOS’s free figure tool, NAAS, to help you prepare publication quality figures: https://journals.plos.org/plosone/s/figures#loc-tools-for-figure-preparation.

NAAS will assess whether your figures meet our technical requirements by comparing each figure against our figure specifications.

# Author comment: Author response to Decision Letter 1

**DOI:** 10.1371/journal.pone.0359337.r002

11 Aug 2026

Submission ID PONE-D-26-25282

Manuscript: "Cardiovascular disease mortality in a Central-West Brazilian capital: a spatial, temporal, and ecological analysis"

Dear Dr Patrick R Stephens, Ph.D

Academic Editor, PLOS One

We appreciate the opportunity to revise our manuscript to adjust it to the PLOS One criteria of publication. We thank the Editor and all three Reviewers for the careful and constructive evaluation of our manuscript.

All suggested corrections have been incorporated into the manuscript. Please note that our following responses are in blue. The page and line numbers referenced throughout this response letter correspond to the tracked-changes version of the revised manuscript. Line numbering may differ in the clean copy.

Sincerely.

Journal Requirements:

When submitting your revision, we need you to address these additional requirements.

1. Please ensure that your manuscript meets PLOS ONE's style requirements, including those for file naming.

Author response: We have reviewed the manuscript against the PLOS ONE formatting templates and confirmed compliance with file naming, reference style, and document structure requirements.

2. You have indicated that data is available from [cepconep.propp@ufms.br.]. Please can we ask you to provide us with a general contact email address for the data requests, so readers can request access in perpetuity. If a general email is not available please provide a link to a website where readers can obtain access to data.

Author response: The email address cepconep.propp@ufms.br is the general institutional contact for the UFMS Research Ethics Committee (CEP/CONEP) and will remain valid for data access requests independent of individual researcher affiliation.

3. Please provide a complete Data Availability Statement in the submission form, ensuring you include all necessary access information or a reason for why you are unable to make your data freely accessible. If your research concerns only data provided within your submission, please write "All data are in the manuscript and/or supporting information files" as your Data Availability Statement.

Author response: Data that are not presented in the article or its Supporting Information files are available upon reasonable request. Due to the presence of sensitive information (residential address) that could compromise participant confidentiality, these data cannot be made publicly available. Requests for access should be directed to the research team and the UFMS Research Ethics Committee (CEP/CONEP-UFMS, cepconep.propp@ufms.br).

Any person who wishes to receive authorisation must: (i) be affiliated with the research team responsible for the project or be accepted as a collaborator; (ii) present a detailed research project together with approval by an appropriate Brazilian institutional research ethics committee; (iii) provide a clear data plan restricted to the objectives of the proposed study and a summary of the analysis plan intended to guide the extraction of the relevant set of records and variables; (iv) sign terms of responsibility regarding the access and use of data.

4. We note that Figure(s) 2, 3 in your submission contain [map/satellite] images which may be copyrighted. All PLOS content is published under the Creative Commons Attribution License (CC BY 4.0), which means that the manuscript, images, and Supporting Information files will be freely available online, and any third party is permitted to access, download, copy, distribute, and use these materials in any way, even commercially, with proper attribution. For these reasons, we cannot publish previously copyrighted maps or satellite images created using proprietary data, such as Google software (Google Maps, Street View, and Earth). For more information, see our copyright guidelines: http://journals.plos.org/plosone/s/licenses-and-copyright.

We require you to either (1) present written permission from the copyright holder to publish these figures specifically under the CC BY 4.0 license, or (2) remove the figures from your submission:

Author response: The maps in Figures 2 and 3 were not created using proprietary software or copyrighted base maps such as Google Maps/Earth. They were generated in R (version 4.5.1, package ggplot2/sf) using publicly available shapefiles provided by the Municipal Geoprocessing System (Sistema Municipal de Geoprocessamento, SIMGEO), a public geospatial database maintained by the Campo Grande City Hall (Secretaria Municipal de Meio Ambiente e Desenvolvimento Urbano, SEMADUR) under Municipal Decree No. 9,520/2006, freely available at https://www.campogrande.ms.gov.br/simgeo. As a Brazilian governmental open-data platform, these shapefiles are public domain administrative data, not copyrighted material, and their use and redistribution under CC BY 4.0 does not infringe any third-party rights.

We have added the following caption/acknowledgment to the figures: "Map was made in R version 4.5.1 (package ggplot2) using shapefiles from SIMGEO, Campo Grande City Hall (SEMADUR)."

5. If the reviewer comments include a recommendation to cite specific previously published works, please review and evaluate these publications to determine whether they are relevant and should be cited. There is no requirement to cite these works unless the editor has indicated otherwise.

Author response: Okay.

6. Please review your reference list to ensure that it is complete and correct. If you have cited papers that have been retracted, please include the rationale for doing so in the manuscript text, or remove these references and replace them with relevant current references. Any changes to the reference list should be mentioned in the rebuttal letter that accompanies your revised manuscript. If you need to cite a retracted article, indicate the article’s retracted status in the References list and also include a citation and full reference for the retraction notice.

Author response: We have reviewed the full reference list against the Retraction Watch Database and Crossref to confirm the status of all cited works. No retracted articles were identified among our references.

Reviewers' comments:

General Response to the Editor and Reviewers

In the course of preparing this revision, we identified that the mortality dataset originally provided by the Campo Grande Municipal Health Department for this study included deaths of individuals who, despite being registered as occurring within the city (place of occurrence), were not residents of Campo Grande (place of residence). As the study's stated population was residents of Campo Grande, we corrected this by applying a residency filter based on the municipality of residence field recorded on each death certificate, retaining only confirmed Campo Grande residents. This correction reduced the analytic sample from 9,792 to 8,148 cardiovascular deaths.

Because this correction affects the numerator of all mortality rates and proportions reported throughout the manuscript, we have recalculated the entire analysis, including Table 1, Table 2, all figures, and all spatial analyses, using the corrected dataset. We have also taken this opportunity to verify and, where necessary, correct the geocoding procedure underlying the spatial analyses, and to expand several analyses in response to the Reviewers' comments below. Where our responses to individual comments below reference figures that differ from those in the original submission, this reflects the correction described here, rather than an independent error specific to that comment.

Reviewer #1:

We sincerely thank Reviewer #1 for the careful and constructive evaluation of our revised manuscript.

Introduction:

It is recommended that, at the end of the introduction, you state more clearly whether the study’s objective was achieved, in accordance with the journal’s guidelines.

We thank the Reviewer for this observation. A statement clarifying the study's objective was added at the end of the Introduction (page 4, lines 86–89).

Results:

It is suggested that the text clarify that information regarding acute myocardial infarction is not presented in tables and/or figures, thereby making it easier for the reader to understand.

Author response: We thank the Reviewer for this observation. We have clarified in the Results section that acute myocardial infarction data are detailed only in S1 Fig, and that subsequent tables and figures report cardiovascular mortality at the disease-group level (page 15, lines 370–373): “...detailed diagnosis-level distributions are presented in Supporting Information Fig S1, as subsequent analyses in this manuscript are reported at the disease-group level (IHD, HF, ARR, CMP, INF) rather than by individual ICD-10 diagnosis.”

When noting that most deaths occurred among men, it is recommended to list the diseases with the highest incidence in descending order, thereby facilitating the organization and interpretation of the results.

Author response: We thank the Reviewer for this observation. The order of the disease list was changed to descending order of magnitude (page 17, lines 385–393).

There was a discrepancy between the figures presented in the text and in Table 1 regarding the race/ethnicity variable. While the text indicates that individuals of mixed race accounted for 41.6% of deaths, Table 1 shows a percentage of 41.7%. This information needs to be revised.

Author response: We thank the Reviewer for this important observation. In the course of this revision, we identified and corrected an error in the study's residency inclusion criteria: a subset of records corresponding to non-residents of Campo Grande had inadvertently been retained in the original dataset (see the General Response to the Editor and Reviewers, which details this correction). Following this correction, both the manuscript text and Table 1 were fully recalculated using the corrected dataset of confirmed Campo Grande residents (N=8,148). Mixed/Pardo individuals now account for 40.0% of deaths (3,260/8,148), and this figure is consistent between the text and Table 1.

An inconsistency was also found in the data on educational attainment. The text states that 48.8% of deaths were among those with a primary school education, while Table 1 shows a figure of 51.8%. Verify the figures and correct the discrepancy.

Author response: We thank the Reviewer for this important observation. As explained in our General Response above, the entire dataset was recalculated following correction of the residency inclusion criteria. Using the corrected dataset (N=8,148), individuals with primary education account for 47.8% of deaths (3,893/8,148), consistent between the text and Table 1.

In Table 1, it is important to include the percentages of disease groups relative to the total number of deaths, to facilitate comparison among categories and provide a more comprehensive view of the distribution of the events analyzed.

Author response: We thank the Reviewer for this observation. Percentages for each disease group relative to the total number of deaths are included in Table 1 column headers (e.g., IHD: N=6,476, 79.5%).

Reviewer #2:

The study is well designed, complies with the journal's guidelines, and presents novel and relevant evidence. The abstract is appropriate and clearly summarizes the objectives, methods, main findings, and conclusions of the study, accurately reflecting the content of the manuscript. The methodology is appropriate, the analyses are well conducted, and the findings make a valuable contribution to the existing literature. The results are robust and are presented clearly and objectively through well-designed figures, graphs, and tables that are easy to understand and effectively support the study's findings. I highly recommend this manuscript for publication.

Author response: We sincerely thank Reviewer #2 for the careful and constructive evaluation of our revised manuscript.

Reviewer #3:

We sincerely thank Reviewer #3 for the careful and constructive evaluation of our revised manuscript.

Introduction

Point 1. Lines 58–59. It’s important to elaborate on the magnitude of the problem within the study setting by including specific epidemiological data on cardiovascular diseases in Campo Grande—such as mortality rates or the proportion of cardiovascular disease deaths relative to total deaths—and comparing the situation in Campo Grande with that of the Central-West region and Brazil as a whole, to demonstrate whether the indicators for the capital exceed state and national averages.

Author response: We thank the Reviewer for this valuable suggestion. We have expanded the Introduction to include specific epidemiological data contextualising the magnitude of CVD mortality in the study setting relative to state, regional, and national indicators (page 4, lines 61–66): “In 2021, the age-standardised CVD mortality rate in Mato Grosso do Sul (170.9 per 100,000) exceeded the national average (162.2 per 100,000) [3]. At the municipal level, large municipalities (>300,000 inhabitants) in the Central-West region recorded lower CVD mortality than the national average for equivalent municipalities in 2016–2018 (158 vs. 178 per 100,000) [7], although this reduction has been shown to be slower and more heterogeneous than in the South and Southeast [7].” We opted not to present a specific mortality rate or proportion of deaths for Campo Grande itself in the Introduction, as this figure constitutes a primary finding of the present study and is reported in the Results section.

Point 2. Lines 62–64. The introduction states that Campo Grande presents a particularly relevant setting for the study due to factors such as its location, demographic flows, and urbanization; however, I suggest describing how these factors specifically influence cardiovascular disease mortality in the study area, thereby justifying this investigation.

Author response: We thank the Reviewer for this observation. We have expanded the Introduction to explain how the cited factors (border location, demographic flows, and urbanisation) are mechanistically linked to cardiovascular risk, based on prior literature (page 5, lines 73–77): “...rapid urbanisation from the 2000s onward [8], factors previously associated with increased cardiovascular risk in Brazil through lifestyle changes, rising exposure to risk factors, and disparities in healthcare access [4]. This border position is additionally associated with significant population mobility, which may further compound heterogeneity in cardiovascular risk exposure and healthcare-seeking patterns.” We note that the specific influence of these factors on CVD mortality in Campo Grande itself is a primary aim of the present investigation and is therefore addressed in the Results and Discussion, rather than anticipated in the Introduction.

Point 3. Line 65. In addition to the role of primary health care, it is important to include information regarding cardiovascular disease surveillance as addressed within the Unified Health System (SUS) by major Brazilian public health policies.

Author response: We thank the Reviewer for this observation. We have added information on a major Brazilian public health policy for cardiovascular risk factor surveillance within the SUS (page 5, lines 77–80): “This period of urbanisation coincided with the expansion of primary health care services [9], which, within the Unified Health System (Sistema Único de Saúde, SUS), included public policies to register, monitor, and treat patients with hypertension and diabetes mellitus, such as the HIPERDIA program.”

Methods

Point 4. Lines 99–105. In the methods section, clarify the inclusion and exclusion criteria for death records regarding the municipality of residence, incomplete records, and potential duplicates. State how deaths with ill-defined causes or records with missing information were handled.

Author response: We thank the Reviewer for the observation. Campo Grande has consistently ranked among the Brazilian state capitals with the highest data completeness for the Mortality Information System (SIM). SIM is fed by a standardised Death Certificate, subject to a multi-step validation and consolidation process before local, state, and federal aggregation, supported nationally by active death-search and vital registration linkage initiatives (Rebouças et al. 2025). Specifically, Campo Grande has consistently ranked among the state capitals with the lowest proportion of ill-defined causes of death in the national Garbage Codes Monitoring Panel (DAENT/SVSA, Brazilian Ministry of Health) (Brasil, 2026), and presents official sub-registration and subnotification rates for deaths of 0.20% and 0.09%, respectively, both well below national averages (3.40% and approximately 1%) (IBGE, 2024); no additional redistribution algorithm (e.g., as applied by the Global Burden of Disease study) was therefore applied in the present analysis.

Regarding inclusion criteria, death records were initially extracted by the Campo Grande Municipal Health Department based on the place of occurrence of death; residency status was subsequently verified using the municipality of residence field recorded on each death certificate, and only deaths of confirmed Campo Grande residents were retained for analysis, resulting in the exclusion of 1,644 records corresponding to non-residents (see General Response at the beginning of this point-by-point response letter). No records were excluded due to missing or incomplete information; missing data for each variable were instead quantified and are reported in the characterisation of the study population (Table 1), as pre-specified in the analysis plan. Regarding duplicate records, death certificates in Brazil are serialised documents whose issuance is centrally controlled by the Ministry of Health, making duplicate records highly unlikely; no duplicate records were identified in the dataset.

As detailed in our response to Point 7, all cardiovascular deaths that proceeded to the geocoding stage were successfully geocoded, with 88.4% matched to exact street addresses, further supporting the completeness and precision of the underlying data.

Rebouças, P., Alves, F. J., Ferreira, A., Marques, L., Guimarães, N. S., Souza, G. R. D., ... & Barreto, M. L. (2025). Avaliação da qualidade do Sistema Brasileiro de Informações sobre Mortalidade (SIM): uma scoping review. Ciência & Saúde Coletiva, 30, e08462023.

Instituto Brasileiro de Geografia e Estatística (IBGE). Estimativas de sub-registro de nascimentos e óbitos 2024. Rio de Janeiro: IBGE; 2026. Disponível em: https://www.ibge.gov.br/estatisticas/sociais/populacao/26176-estimativa-do-sub-registro.html

Brasil. Ministério da Saúde. SVSA/DAENT. Painel de Monitoramento da Mortalidade por Causas Básicas Inespecíficas ou Incompletas (Garbage Codes) [Internet]. Brasília: MS; [citado 2026]. Disponível em: https://svs.aids.gov.br/daent/centrais-de-conteudos/paineis-de-monitoramento/mortalidade/codigos-garbage/

Point 5. Lines 92–93. The 74 neighborhoods were treated as units of analysis for the ecological study; however, it is not stated whether they exhibit population stability, whether neighborhoods with small populations were aggregated, or whether the rates were calculated directly or subjected to smoothing.

Author response: We thank the Reviewer for this observation. We have addressed each of the three points raised:

Population stability: Neighbourhood-level population was not assumed stable. Annual population estimates for each of the 74 neighbourhoods (2013–2022) were obtained by geometric interpolation between the 2010 and 2022 Population Censuses, applying neighbourhood-specific growth rates, following the same intercensal estimation method used by IBGE at the municipal level. These estimates were proportionally calibrated (pro-rated) to match official annual municipal population totals, since the sum of the 74 neighbourhood populations represented 94.4–98.4% of the municipal total in each year, the residual being attributable to rural and peri-urban areas outside the formal neighbourhood boundaries. This methodology has been added to the Methods section (page 7, lines 136–144).

Aggregation of small-population neighbourhoods: Neighbourhoods with small populations were not aggregated; all 74 official neighbourhoods, as defined by SIMGEO, were retained as independent units of analysis. Instead of aggregation, instability arising from small case counts in low-population neighbourhoods was addressed through Bayesian spatial smoothing (see response below).

Smoothing of rates: Crude neighbourhood-level mortality rates were smoothed using a Besag-York-Mollié (BYM) Bayesian hierarchical model, implemented via the CARBayes package in R, which combines spatially structured and unstructured random effects to stabilise rate estimates in areas with small numbers of events or low population, while preserving genuine spatial variation in mortality risk. Convergence of the BYM model was assessed by comparing posterior estimates across independent Markov Chain Monte Carlo (MCMC) runs with different random seeds; the correlation between smoothed rate estimates from two independent runs was consistently greater than 0.999, indicating stable convergence. Both crude and smoothed rates are presented in Fig 2 for comparison, and smoothed rates were used for the LISA cluster analysis (Fig 4) to reduce the influence of estimation instability on cluster detection. This methodology has been added to the Methods section (page 13, lines 285–298).

Point 6. Lines 216–217. It is recommended to explicitly present the formulas used to calculate mortality rates, indicating the numerator and denominator and whether calculations were performed at the neighborhood level. Was spatial standardization of the rates applied?

Author response: We thank the Reviewer for this observation. The formula for mortality rate calculations, indicating numerator and denominator, is presented in the Descriptive analysis subsection (page 10, lines 213–215): “Crude mortality rates were calculated for each year, disease group, and sex by dividing the number of deaths by the corresponding mid-year population estimate, expressed per 100,000 inhabitants.”

The formula specific to neighbourhood-level rates is presented in the Spatial Analysis subsection (page 13, lines 285–288): “Neighbourhood-level cardiovascular mortality rates were calculated as the number of deaths accumulated over the study period (2013–2022) divided by the corresponding neighbourhood person-years (the sum of the annual calibrated population estimates described in the “Data sources” section), expressed per 100,000 inhabitants.”

Regarding spatial standardisation, we have clarified that neighbourhood-level rates were not age-standardised, owing to the unavailability of age-stratified population data at this spatial resolution (page 13, lines 296–298); both crude and Bayesian-smoothed (BYM) rates are reported for comparison, as detailed in our response to Point 5.

Point 7. Line 225. For spatial analysis, it is important to present certain essential information, such as the address unit used and how unlocated addresses were handled.

Author response: We thank the Reviewer for this observation. We have clarified the geocoding procedure in the Methods section. Deaths were geocoded in R using the ArcGIS World Geocoding Service via the tidygeocoder package, with a hierarchical address-matching strategy, prioritising, in order: exact street address, street-level interpolation, neighbourhood centroid, and municipality centroid. All cardiovascular deaths among confirmed Campo Grande residents (n = 8,148) proceeded to geocoding. Geocoding precision was as follows: exact address, 91.7% (n = 7,469); street level, 3.2% (n = 257); neighbourhood/city centroid, 2.3% (n = 184); postal code/PO box, 2.1% (n = 172); region/state level, 0.8% (n = 65); and sub-address, 0.01% (n = 1). Of the small proportion of cases initially unmatched to a neighbourhood polygon following geocoding (n = 104, 1.3%), the large majority corresponded to genuinely rural or peri-urban addresses outside the formal neighbourhood boundaries (e.g., the rural district of Anhanduí) and were excluded from the neighbourhood-level spatial analysis; a smaller number were resolved using the self-reported neighbourhood field recorded independently on the death certificate at the time of registration, cross-validated against the official neighbourhood boundaries. In total, 69 cases (0.85% of confirmed residents) were excluded from the neighbourhood-level spatial analysis due to rural location or unresolved address matching; these cases were retained in all non-spatial analyses (e.g., temporal and sex-specific analyses). This is acknowledged as a limitation in the Discussion.

Point 8. Lines 235–249. Specify the parameters used in the Kernel Density Estimation (KDE) analyses, as well as the criteria for the Global Moran's I and LISA analyses.

Author response: We thank the Reviewer for the observation. Parameters and criteria for the KDE and spatial autocorrelation analyses have been explicitly stated in the Methods (page 14, lines 307–309): KDE was performed in R using a Gaussian kernel with a fixed bandwidth of 800 metres and spatial resolution of 50 metres per pixel; LISA quadrants were classified based on deviations from the mean of the variable and its spatial lag; and the Global Moran's I and LISA analyses used a Queen contiguity matrix with row-standardised (W) weights, with statistical significance for the Global Moran's I assessed via 999 Monte Carlo permutations and a significance threshold of α = 0.05 applied throughout.

Results

Point 9. Results. It is recommended to clarify which of the presented results correspond primarily to crude or standardized rates, so as to avoid potential misinterpretations.

Author response: We thank the Reviewer for this observation. We have revised the manuscript throughout to explicitly distinguish between crude and adjusted statistics, using two distinct types of adjustment as appropriate: age-standardisation (temporal analyses) and Bayesian spatial smoothing (neighbourhood-level analyses). Specifically: (i) the opening paragraph of the Results now reports absolute case counts, which require no such distinction; (ii) temporal trends are reported for both crude and age-standardised mortality rates (ASMR), presented side by side in Supporting Information Fig S2, with all annual percentage change (APC) and Prais-Winsten estimates in Table 2 based on crude rates, as now stated explicitly; (iii) the sex-specific incidence rate ratio is now labelled as a crude IRR; (iv) neighbourhood-level choropleth maps (Fig 2) explicitly present crude and BYM-smoothed rates side by side for direct comparison, rather than age-standardisation, owing to the unavailability of age-stratified population data at the neighbourhood level (also addressed in our response to Point 6); and (v) Kernel Density Estimation (Fig 3) is explicitly labelled as a density estimate rather than a population-based rate, to avoid any ambiguity with the rate-based analyses.

Point 10. Lines 266–271. Some results describe frequencies but do not report the total number of records included, which could indicate any exclusions made.

Author response: We thank the Reviewer for this observation. We have revised the Results section throughout to report the denominator (n) alongside all percentages describing frequencies, including race/ethnicity, education level, place of death, and age distribution.

Point 11. Table 1. I suggest presenting information on missing data for the main variables analyzed—age, sex, race, education, marital status, and place of occurrence—given that these are secondary data from the Mortality Information System (SIM).

Author response: We thank the Reviewer for this suggestion. Information on missing data has been added to Table 1 for all main variables (sex, age group, race/ethnicity, education level, marital status, and place of death), reported as n (%) for each variable.

Discussion

Point 12. The first paragraph repeats the results. I recommend making the opening section of the discussion more interpretive, highlighting novel findings and their implications for public health.

Author response: We thank the Reviewer for this suggestion. We have revised the opening paragraph of the Discussion to reduce repetition of specific figures already presented in the Results, replacing them with a more interpretive framing that highlights the study's three most novel and policy-relevant findings and their public health implications (page 28–29, lines 515–523).

Point 13. The association between increased mortality after 2019 and the COVID-19 pandemic is plausible; however, the discussion presents a temporal relationship that might imply causality. It is recommended to emphasize that the study does not allow for directly attributing the increase to the consequences of COVID-19.

Author response: We thank the Reviewer for this important observation. We agree that temporal coincidence does not establish causality. We have revised the Discussion to make this limitation explicit (page 31, lines 582-585): “However, this study's ecological, observational design does not permit direct attribution of this acceleration to COVID-19, and this temporal association should be interpreted as hypothesis-generating rather than causal.” This statement precedes our existing discussion of potential attribution bias in cardiovascular cause-of-death coding during the pandemic period, which further supports a cautious interpretation of this finding.

Point 14. The study highlights the rise in ischemic heart disease (IHD) mortality in contrast to the national decline. It is recommended to discuss potential regional factors, such as varying epidemiological transitions, population aging, the prevalence of risk factors, and inequalities in cardiovascular care—including access to the cardiovascular disease care pathway.

Author response: We thank the Reviewer for this valuable suggestion. We have expanded the Discussion to address potential regional factors underlying the divergence between the increasing IHD mortality observed in Campo Grande and the national declining trend, including the region's lagging epidemiological transition, demographic factors related to population ageing and migration, and documented national-level disparities in access to cardiac diagnostic testing, interventional cardiology services, and their association with ischaemic heart disease mortality (page 29, lines 528-541).

Point 15. Home-based mortality due to IHD is a significant finding; however, I recommend discussing key aspects of this scenario, such as the availability of and access to emergency services, the time between symptom onset and care (response time), the organization of the emergency care network, and the role of Primary Health Care in identifying cardiovascular risk.

Author response: We thank the Reviewer for this valuable suggestion. We have expanded the Discussion to address each of the aspects raised: emergency service availability and access, prehospital response time, the organisation of the emergency care network, and the role of Primary Health Care in cardiovascular risk identification, drawing on national Brazilian evidence on prehospital delay, geographic access barriers, telemedicine-supported emergency networks, and the existing HIPERDIA programme referenced in the Introduction (page 30, lines 548-571).

Point 16. It is important to strengthen the discussion regarding study limitations—potentially including the ecological fallacy—by noting that neighborhood-level associations do not necessarily reflect individual-level relationships.

Author response: We thank the Reviewer for this observation. We have strengthened the Limitations paragraph to explicitly name and define the ecological fallacy, clarifying that neighbourhood-level associations observed in this study do not necessarily reflect individual-level relationships (page 33, lines 622-625): “The ecological design precludes individual-level causal inference and is subject to the ecological fallacy, whereby associations observed at the neighbourhood level do not necessarily reflect relationships that would be observed at the individual level; results may additionally be sensitive to the choice of spatial unit (modifiable areal unit problem).”

Point 17. Include implications for health policies, particularly practical applications, especially given that this is a municipal-level study. I suggest addressing the following aspects: strengthening cardiovascular epidemiological surveillance, integrating surveillance with Primary Health Care, prioritizing areas with the highest mortality burden, and implementing prevention and early diagnosis strategies.

Author response: We thank the Reviewer for this observation. These policy implications were incorporated into the Conclusion, addressing each of the four aspects suggested: strengthening cardiovascular epidemiological surveillance, integrating surveillance with Primary Health Care, prioritising areas with the highest mortality burden, and implementing targeted prevention and early diagnosis strategies (page 34, lines 644-659).

Attachment Submitted filename: Response-to-Reviewers_R1_PLOS-One_v1_Joao.pdf

# Peer review: Decision Letter 1

**DOI:** 10.1371/journal.pone.0359337.r003

**Authors:** Patrick R Stephens

13 Sep 2026

Cardiovascular disease mortality in a Central-West Brazilian capital: a spatial, temporal, and ecological analysis

PONE-D-26-25282R1

Dear Dr. Falcão de Oliveira,

We’re pleased to inform you that your manuscript has been judged scientifically suitable for publication and will be formally accepted for publication once it meets all outstanding technical requirements.

Within one week, you’ll receive an e-mail detailing the required amendments. When these have been addressed, you’ll receive a formal acceptance letter and your manuscript will be scheduled for publication.

An invoice will be generated when your article is formally accepted. Please note, if your institution has a publishing partnership with PLOS and your article meets the relevant criteria, all or part of your publication costs will be covered. Please make sure your user information is up-to-date by logging into Editorial Manager at Editorial Manager® and clicking the ‘Update My Information' link at the top of the page. For questions related to billing, please contact billing support.

If your institution or institutions have a press office, please notify them about your upcoming paper to help maximize its impact. If they’ll be preparing press materials, please inform our press team as soon as possible -- no later than 48 hours after receiving the formal acceptance. Your manuscript will remain under strict press embargo until 2 pm Eastern Time on the date of publication. For more information, please contact onepress@plos.org.

Kind regards,

Patrick R Stephens, Ph.D.

Academic Editor

PLOS One

Additional Editor Comments (optional):

Reviewers' comments:

Reviewer's Responses to Questions

**Comments to the Author**

Reviewer #1: All comments have been addressed

Reviewer #3: All comments have been addressed

**********

2. Is the manuscript technically sound, and do the data support the conclusions?

Reviewer #1: Yes

Reviewer #3: Yes

**********

3. Has the statistical analysis been performed appropriately and rigorously?

Reviewer #1: Yes

Reviewer #3: Yes

**********

4. Have the authors made all data underlying the findings in their manuscript fully available?

The PLOS Data policy

Reviewer #1: Yes

Reviewer #3: Yes

**********

5. Is the manuscript presented in an intelligible fashion and written in standard English?

Reviewer #1: Yes

Reviewer #3: Yes

**********

Reviewer #1: After reviewing the revised version, it was determined that the requested corrections and adjustments were made satisfactorily, addressing the comments provided. The manuscript is adequate in terms of methodological, structural, and textual aspects, and there are no outstanding issues that would prevent its publication. Therefore, I consider the article to be properly corrected and suitable for publication.

Reviewer #3: The authors have addressed all corrections suggested during the review process by making the necessary adjustments to the manuscript.

All 17 items have been addressed, and the relevant points have been adjusted across the various sections of the manuscript.

**********

what does this mean?). If published, this will include your full peer review and any attached files.

If you choose “no”, your identity will remain anonymous but your review may still be made public.

**Do you want your identity to be public for this peer review?**  For information about this choice, including consent withdrawal, please see our Privacy Policy

Reviewer #1: No

Reviewer #3: No

**********

# Editor report: Acceptance letter

**DOI:** 10.1371/journal.pone.0359337.r004

**Authors:** Patrick R Stephens

PONE-D-26-25282R1

PLOS One

Dear Dr. Falcão de Oliveira,

I'm pleased to inform you that your manuscript has been deemed suitable for publication in PLOS One. Congratulations! Your manuscript is now being handed over to our production team.

At this stage, our production department will prepare your paper for publication. This includes ensuring the following:

* All references, tables, and figures are properly cited

* All relevant supporting information is included in the manuscript submission,

* There are no issues that prevent the paper from being properly typeset

You will receive further instructions from the production team, including instructions on how to review your proof when it is ready. Please keep in mind that we are working through a large volume of accepted articles, so please give us a few days to review your paper and let you know the next and final steps.

Lastly, if your institution or institutions have a press office, please let them know about your upcoming paper now to help maximize its impact. If they'll be preparing press materials, please inform our press team within the next 48 hours. Your manuscript will remain under strict press embargo until 2 pm Eastern Time on the date of publication. For more information, please contact onepress@plos.org.

You will receive an invoice from PLOS for your publication fee after your manuscript has reached the completed accept phase. If you receive an email requesting payment before acceptance or for any other service, this may be a phishing scheme. Learn how to identify phishing emails and protect your accounts at https://explore.plos.org/phishing.

If we can help with anything else, please email us at customercare@plos.org.

Thank you for submitting your work to PLOS One and supporting open access.

Kind regards,

PLOS One Editorial Office Staff

on behalf of

Dr. Patrick R Stephens

Academic Editor

PLOS One
